# Molecular Characterization and Expression of a Novel Alcohol Oxidase from *Aspergillus terreus* MTCC6324

**DOI:** 10.1371/journal.pone.0095368

**Published:** 2014-04-21

**Authors:** Mitun Chakraborty, Manish Goel, Somasekhar R. Chinnadayyala, Ujjwal Ranjan Dahiya, Siddhartha Sankar Ghosh, Pranab Goswami

**Affiliations:** Department of Biotechnology, Indian Institute of Technology Guwahati, Assam, India; University of California, San Diego, United States of America

## Abstract

The alcohol oxidase (AOx) cDNA from *Aspergillus terreus* MTCC6324 with an open reading frame (ORF) of 2001 bp was constructed from *n*-hexadecane induced cells and expressed in *Escherichia coli* with a yield of ∼4.2 mg protein g^−1^ wet cell. The deduced amino acid sequences of recombinant rAOx showed maximum structural homology with the chain B of aryl AOx from *Pleurotus eryngii*. A functionally active AOx was achieved by incubating the apo-AOx with flavin adenine dinucleotide (FAD) for ∼80 h at 16°C and pH 9.0. The isoelectric point and mass of the apo-AOx were found to be 6.5±0.1 and ∼74 kDa, respectively. Circular dichroism data of the rAOx confirmed its ordered structure. Docking studies with an *ab-initio* protein model demonstrated the presence of a conserved FAD binding domain with an active substrate binding site. The rAOx was specific for aryl alcohols and the order of its substrate preference was 4-methoxybenzyl alcohol >3-methoxybenzyl alcohol>3, 4-dimethoxybenzyl alcohol > benzyl alcohol. A significantly high aggregation to ∼1000 nm (diameter) and catalytic efficiency (*k_cat_/K_m_*) of 7829.5 min^−1^ mM^−1^ for 4-methoxybenzyl alcohol was also demonstrated for rAOx. The results infer the novelty of the AOx and its potential biocatalytic application.

## Introduction

The alcohol oxidase (AOx) enzymes (Alcohol: O2 Oxidoreductase; EC 1.1.3.x) catalyze the oxidation of various alcohol substrates to the corresponding carbonyl compounds with a concomitant release of hydrogen peroxide. Over the last decade these flavoenzymes have attracted wide attention for their potential industrial applications due to the fact that they can catalyze the oxidation of alcohol substrates irreversibly and selectively, and they do not require any external co-factors for the catalysis since the cofactor, which is flavin or their derivative, is avidly linked to the protein matrix of these redox enzymes. Moreover, these groups of enzymes are easily available in nature because various aerobic microorganisms produce them and thus offer the scope for their large scale production using suitable bioreactors. The potential areas of application of AOxs are biocatalytic synthesis of flavors, fragrances, optically pure compounds, biosensors, and to a limited extent the bioremediation of hydrocarbon contamination [12], [20], [6], [31]. AOxs may be categorized broadly into four different groups namely, (1) Short chain alcohol oxidase (SCAO) (2) Long chain alcohol oxidase (LCAO) (3) Aromatic alcohol oxidase (AAO) and (4) Secondary alcohol oxidase (SAO) [22]. The sources reported for these enzymes are mostly bacteria, yeast, fungi, plant, insect and mollusks.

AOx has been reported as one of the inducible enzymes formed during microbial uptake of aliphatic hydrocarbons and the microbial sources described in reports are predominantly yeast species [7], [18], [42], [8], [21]. There are only few reports on hydrocarbon grown filamentous fungi as the source organisms for AOx and among them *Cladosporium* species [13], *Aspergillus* species [38], and YR-1 strain [33] are well described. However, all these reports largely deal with long chain (or fatty) AOx. Interestingly, our previous work on filamentous fungi, *Aspergillus terreus* MTCC6324 revealed that the strain exhibits all the four categories of AOx activity in the cells during the growth on hydrocarbon substrates [22]. The different AOx activities were located in the microsomal membrane of the cell and all activities were confined to highly aggregated protein particles purified from the cell homogenate by chromatographic means [23]. However, characterizations of the individual AOx enzyme following the conventional purification protocols were a challenge posed by the very high aggregating tendency of the isolated complex enzyme assembly and low protein yields.

**Figure 1:**
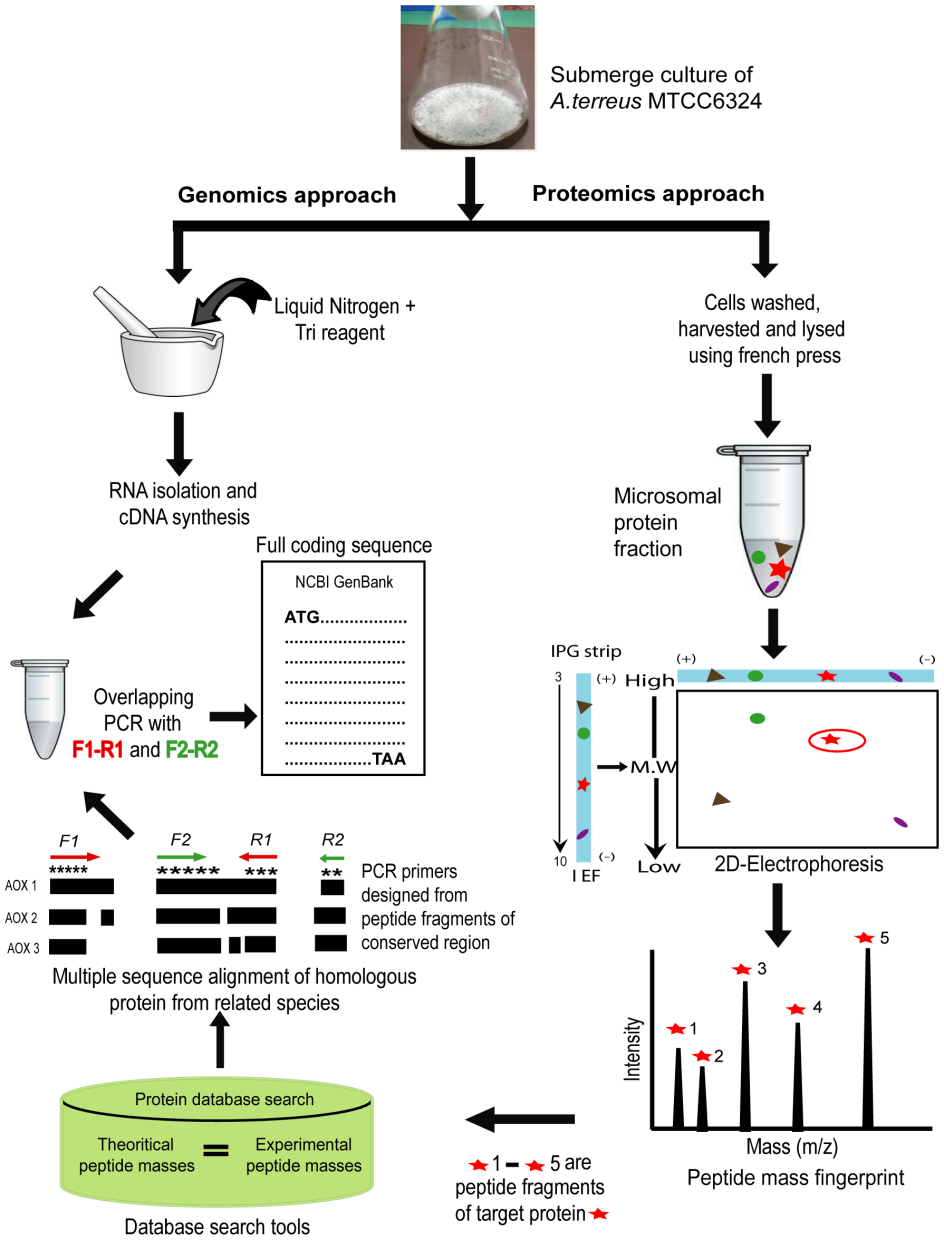
An overall schematic diagram highlighting the proteomics and genomics approach undertaken to characterize AOx cDNA. Target protein is marked in red star. AOX 1, AOX 2 and AOX 3 represent the amino acid sequences of different AOxs. F 1, F 2, R 1 and R 2 represent the PCR primers.

Molecular characterization and cloning of *A.terreus* genes for commercially significant enzymes is limited primarily due to lack of its available reliable genome sequence and inadequate information in annotated, non-redundant protein database. The current research work thus utilized a combinatorial approach of proteomics and genomics to elucidate a full length *A.terreus* MTCC6324 cDNA of AOx (**Fig. 1**). Here we report for the first time the molecular characteristics, cDNA cloning, and expression of an AOx gene from *n*-hexadecane induced *A.terreus* MTCC6324. Heterologous cloning and expression of the characterized gene in *E.coli* leads to the production of the apo-AOx enzyme, which was subsequently transformed into a functionally active AOx protein following an *in-vitro* FAD reconstitution approach. In a parallel work, *ab-initio* based rAOx protein structure and function was investigated using the translated amino acid sequence of the characterized gene and molecular docking studies predicted aryl alcohols as the substrates for the enzyme, which were validated by the follow-up wet-lab experiments using the rAOx. The high aggregating nature and kinetic characteristics of the rAOx enzyme were also analyzed and compared with the native enzyme and is reported in the present communication.

## Materials and Methods

### Materials

The culture conditions and maintenance of *A. terreus* MTCC 6324 used in this study were described previously [22]. The organism was cultivated in 500 ml Erlenmeyer flasks containing 2% (v/v) n-hexadecane in 50 ml basal medium of the following compositions (g l^−1^): MgSO_4_.7H_2_O, 0.2; NH_4_NO_3_, 1.0; CaCl_2_, 0.02; KH_2_PO_4_, 1.0; K_2_HPO_4_, 1.0; and yeast extract, 1.0. The pH of the medium was adjusted to 5.8, autoclaved and incubated in static condition after inoculation with fungal mycelia at 28°C. *E.coli* DH5α and BL21 (DE3) were grown as described in Sambrook *et al* [37]. TA cloning was performed using pGEMT Easy cloning kit (Promega, USA) and expression in BL21 (DE3) was carried out using pET28a (+) expression vector (Novagen, Merck, Germany). Standard molecular biology and mass spectrometry kits, fine chemicals and chromatography grade reagents were purchased from Sigma Aldrich (USA). Restriction enzymes were procured from New England Biolabs, USA.

### Methods

#### Isolation of total RNA and preparation of cDNA

Thin, papery, translucent mycelium from four day old static/submerge culture of *A. terreus* MTCC6324 were harvested and washed thoroughly with chilled double distilled water to remove excess of *n*-hexadecane and media components. The cells were pretreated with benzyl chloride [26]. Approximately, 100–200 mg wet weight of mycelium were filtered through a filter paper to remove excess of water and placed in a mortar pestle pre-chilled with liquid nitrogen. All the necessary labwares, including tips, eppendorfs, spatulas and mortar pestle were pretreated with diethylpolycarbonate to minimize the effect of RNase contamination. The cells were homogenized thoroughly with liquid nitrogen in a mortar pestle. 2 ml of TRI reagent (Sigma Aldrich, USA) were immediately added without allowing the homogenized cells to thaw. The cell lysate was passed several times through a 5 ml syringe with needles intact until the cell homogenate becomes viscous and passes easily through the needle. In the subsequent steps of total RNA isolation, manufacturer's protocol for plant RNA isolation was optimized with TRI reagent. The total RNA isolation protocol was optimized for its yield and purity. RNA samples were dissolved in RNase-free water and their concentration and purity were determined through spectroscopy. For comparison, electrophoresis was carried out in 1.5% 3-(*N*-morpholino) propane sulfonic acid (MOPS)/formaldehyde agarose gel [27], [37].

Reverse-transcriptase polymerase chain reaction (RT-PCR) based first strand cDNA synthesis was achieved using random hexamer primer following manufacturer's protocol (RevertAid H Minus first strand cDNA synthesis kit, Fermentas life sciences). The quality of the cDNA was accessed by the amplification of glyceraldehyde 3-phosphate dehydrogenase (GAPDH) as a housekeeping gene with GAPDH specific forward (GAPDH-F) and reverse primers (GAPDH-R) provided with the kit (**Table S1, Sl no. 1 and 2**). The GAPDH housekeeping gene amplification was carried out following kit manufacturer's protocol. The PCR product was analyzed on 0.5% agarose gel to check the quality of the cDNA synthesized.

#### Preparation of *A. terreus* MTCC6324 microsomal protein extracts

*A.terreus* cells were harvested at early stationary phase of its growth in static culture. The harvested mycelium were separated from the culture broth and washed with chilled 50 mM Tris/HCl buffer, pH 8.0. The cells were briskly washed with *n*-hexane to prepare substrate free cell mass following the protocol described by Goswami and Cooney [13]. The cells were macerated in mortar pestle and further homogenized in french press (Constant cell disruption Systems, UK) at 30 kilo pounds per square inch (kpsi). The cell homogenate was processed to isolate the supernatant of the microsomal fraction following the procedure as described in Kumar and Goswami [22]. AOx activity was assayed using horseradish peroxidase based coupled assay method [46].

#### Two-dimensional (2D) gel electrophoresis

Active supernatant from microsomal fraction was dialysed against 50 mM Tris/HCl buffer (pH 8.0) overnight at 4°C. The dialyzed protein was concentrated with poly (ethylene) glycol 6000 until the protein concentration by Bradford assay [3] reached ∼1 mg ml^−1^. 2D gel electrophoresis was performed according to the method described by Gőrg *et al* [11]. 150 µg per 150 µl protein sample was mixed with 250 µl of rehydration solution containing 8 M urea, 2% (w/v) 3-[(3 Cholamidopropyl) dimethylammonio] -1-propanesulfonate (CHAPS), 0.002% (v/v) bromophenol blue (1% w/v stock). DL-Dithiothreitol (DTT) and pH 3–10 IPG buffer or Pharmalyte (GE Healthcare, Sweden) were added just prior to use. Sample with rehydration buffer was reduced and alkylated using ProteoPrep Reduction and Alkylation kit (Sigma, USA) following manufacturer's protocol. Sample was then passively rehydrated onto immobiline dry pH gradient strips (linear pH gradient 3–10, 18 cm, GE Healthcare, Sweden) for 16 h at 20°C. Strips were overlaid with mineral oil to prevent dehydration. The strip was focused at 500 V for 5 h (step and hold), 1000 V for 1 h 30 min (gradient), 10,000 V for 3 h, 10,000 V for 1 h (step and hold) using Ettan IPGphor 3 isoelectric focusing unit (GE Healthcare, Sweden). Focused strip was equilibrated in two steps for reduction and alkylation using ProteoPrep Reduction and Alkylation kit (Sigma Aldrich, USA) following kit's procedure. Strip was then loaded onto a vertical polyacrylamide gel and run in second dimension without stacking gel, using 18 cm×16 cm, 12% polyacrylamide gel. A blotting paper soaked in protein molecular weight marker was loaded onto the gel. The gel was sealed with agarose sealing solution having 0.5% (w/v) agarose (Sigma Aldrich, USA) and 0.002% (v/v) bromophenol blue (1% w/v stock) in 1× sodium dodecyl sulphate (SDS) electrophoresis buffer. Electrophoresis was carried out following standard protocol [24] in vertical electrophoresis unit (SE 600 Ruby, GE Healthcare).

Mass spectrometry compatible silver staining of polyacrylamide gel after second dimension was carried out following the protocol of Chevallet *et al* [5]. The gel was scanned using Image Scanner III (GE Healthcare, Sweden) and analysed using labScan 6.0 software (GE Healthcare, Sweden). Spot detection and excision was done manually.

#### Protein identification from 2D gel spots

Silver stained 2D gel was manually investigated for protein spots, subsequently excised and digested by trypsin following an optimized in-gel digestion protocol of Shevchenko *et al* [40]. Each spots were cut into cubes of approximately 1×1 mm and 500 µl per spot of acetonitrile was used to shrink the gel pieces for 10 min followed by a brief spin. The liquid above the gel pieces were removed and discarded. 30–50 µl of DTT solution (10 mM DTT in 100 mM ammonium bicarbonate) was added to completely submerge the gel pieces and incubated at 56°C for 30 min in a water bath. The tubes were chilled at room temperature (RT) (22°C) and 500 µl of acetonitrile was added to each tube, incubated for 10 min and the liquid was pipetted out. 30–50 µl of iodoacetamide solution (55 mM in 100 mM ammonium bicarbonate) were added to completely cover the gel pieces and incubated in dark at RT for 20 min. The gel pieces were finally dehydrated with acetonitrile and all the liquid was removed. DTT and iodoacetamide solutions were prepared shortly before use. The gel pieces were covered with 50 µl of trypsin buffer [50 mM ammonium bicarbonate containing 10% (v/v) acetonitrile] and incubated on ice for 30 min. 20 µl of freshly prepared trypsin stock solution (20 µg ml^−1^ in trypsin buffer supplemented with 1 mM HCl) was added to the gel samples so as to cover the gel pieces completely and incubated overnight at 37°C. After incubation, all the liquid above the gel pieces were transferred to a fresh sterile tube and gels were again incubated with peptide extraction buffer (1∶2 v/v 5% formic acid/acetonitrile) for 30 min at 37°C in water bath. The peptide extraction solution was removed and combined with the liquid in the previous step.

Target protein identification by Matrix Assisted Laser Desorption Ionization-Time of Flight-Mass Spectrometry (MALDI-TOF-MS) (4800 plus MALDI TOF/TOF Analyzer, AB SCIEX, USA) was carried out for each of the 2D gel spots. Samples for MALDI-TOF-MS was prepared by mixing 6 µl of saturated α-cyano-4-hydroxycinnamic acid matrix (10 mg ml^−1^ of 50% acetonitrile and 0.05% trifluoroacetic acid in 0.2 micron filtered water) with 6 µl of eluted peptide solution for each spot. The samples were prepared immediately and vortexed to make a homogenous solution and 1 µl of the mixture was dropped onto the metal target plate. The samples were air-dried completely. The plate was properly aligned and calibrated using calibration mixture (4700 proteomics analyzer calibration mix, AB SCIEX, USA). Samples for calibration were made according to the manufacturer's instructions. Samples were analyzed using MS mode to obtain the peptide mass fingerprint (pmf) of each 2D spot. For each sample 2D spot, peak list was generated using 4000 series explorer software package (AB SCIEX, USA).

#### Multiple sequence alignment (msa) studies and pmf database search

Multiple amino acid sequence alignment of AOxs from different filamentous fungi and yeast species were done with ClustalX version 2.1 [19] and the data file generated were visualized and analyzed through GeneDoc software tool [30]. Unreviewed hypothetical AOx protein sequence from *A.terreus* NIH2624 (GI: 115437438) was taken as a query sequence in NCBI protein BLAST. Different filamentous fungi and yeast species coding for AOx enzyme, having 60% or more sequence homology with the query were taken for protein msa. Highly conserved amino acid sequence motifs were used for further sequence analysis.

The pmf peak lists generated from each MALDI sample corresponding to each 2D gel spots were analyzed for detectable m/z values. The peptide masses resulting from specific and unspecific cleavage of the protein were matched with the theoretical masses generated from the peptides of unreviewed AOx protein sequence mentioned above.

#### Designing overlapping PCR primer probes to amplify the coding sequence of AOx

Primer probes for full length amplification of *AOx* gene from cDNA of *A.terreus* MTCC6324 were designed based on the peptide sequence information retrieved from the pmf and msa data generated previously. A top down proteomics based gene identification strategy was undertaken to characterize AOx coding sequence from cDNA of *A.terreus* MTCC6324. Two overlapping PCR amplicons covering the entire coding sequence were performed. For the first PCR, the forward primer (**Table S1, Sl no. 3**) was designed from the 5′ end (start codon) of *AOx* coding sequence of *A.terreus* NIH2624 and the reverse primer (**Table S1, Sl no. 4**) was designed based on the internal peptide fragment from pmf in-sync with the conserved region of msa data. The second overlapping PCR amplicon was generated using the forward primer (**Table S1, Sl no. 5**) designed from internal peptide fragment of pmf data corresponding to the conserved region of msa, and reverse primer (**Table S1, Sl no. 6**) designed from 3′ end of *AOx* sequence information of *A.terreus* NIH2624 available in NCBI database.

Both the PCRs were optimized in 50 µl reaction volume using GeneAmp PCR system 9700 (Applied Biosystems, USA) using high fidelity taq DNA polymerase (Bioline, UK). PCR conditions for both the overlapping PCRs were optimized with initial denaturation at 94°C for 5 min; 35 cycles of amplification [a denaturation step at 94°C for 30 sec, annealing at (60°C for first PCR product and 63°C for second PCR product) for 45 sec, initial extension at 72°C for 1.5 min]; final extension at 72°C for 10 min.

#### Characterization of full length ORF of AOx from overlapping PCR product

Each PCR fragments were subsequently cloned into TA cloning vector (pGEM-T Easy, Promega, USA) and individual DNA sequencing was performed (3130xl Genetic Analyzer, Applied Biosystems). *Bgl*II and *Nde*I as unique restriction enzyme sites present in the overlapping region of both the PCR fragments and in the pGEMT vector backbone, respectively, were chosen for checking the proper orientation of the cloned fragments. First PCR product harboring the start codon of *AOx* open reading frame was ligated with second PCR fragment harboring the stop codon, at the common restriction site, thus confirming the full length functional clone of AOx from *A.terreus* MTCC6324. The full length clone of AOx in TA vector was confirmed through primer walking of both DNA strands (3130xl Genetic Analyzer, Applied Biosystems).

#### *In-silico* sequence analysis, protein structure prediction and molecular docking

Contigs from primer walking data were aligned using DNA Baser sequence assembly software package (Heracle BioSoft SRL, Italy) which predicted the full length coding sequence of *AOx*. Nucleotide sequence translation was performed by Sixpack application tools (European Molecular Biology Open Software Suite, EMBOSS). Sequence homology at protein and nucleotide level with un-reviewed sequence information of AOx (*A.terreus* NIH strain) were analysed using ClustalX version 2.1. Conserve domain search was performed through NCBI Conserved Domains tool [28] to check for conserved N-terminal Rossmann fold motif in our deduced amino acid sequences (**data not shown**).

A composite approach combining threading, *ab-initio* modeling and atomic level structural refinement was adopted using Iterative Threading Assembly Refinement (I-TASSER) server [34]. Translated amino acid sequence was given as a query and I-TASSER identified suitable templates from PDB using threading algorithms and predicted top five 3D models. Models were ranked according to their confidence score (C-score), template modeling score (TM-score) and root mean square deviation (RMSD) values. The top ranking model with a C-score closer to 2 in range of −5.0 to 2.0, TM-score greater than 0.5 and a rationale RMSD value was selected for further structural validation. The best predicted model was analyzed for unusual amino acid content through Ramachandran plot in PROCHEK (European Molecular Biology Laboratory-European Bioinformatics Institute server) [25]. Templates used by I-TASSER to predict the model were also analyzed for its stereochemical quality

To further validate and investigate the sequence to structure to function paradigm, the modeled apoenzyme was docked with its co-factor FAD (from PDB id: 3FIM) using Molegro virtual docker (MVD) (Molegro, CLC bio, Denmark) [41]. Best conformation of FAD with its binding cavity was elucidated by manually investigating each cavity for its conserved interacting residues abiding the well established Rossmann fold architecture (GXGXXG motif, X = any amino acid residue) present at the N-terminal region [32]. 3D superimposition of FAD from our docking studies was performed with that of the FAD bound to the crystal structure of 3FIM. *In-silico* docking with different aromatic alcohols was performed to get an overall assessment of the active site known for catalytic reaction. Moldock score based binding energies were evaluated and further validated through wet lab enzyme kinetic studies.

#### Cloning AOx into *E.coli* expression vector

The AOx gene cloned into TA cloning vector was PCR amplified using forward (**Table S1, Sl no. 7**) and reverse (**Table S1, Sl no. 8**) primers and ligated to expression plasmid pET28a (+) (Novagen, Merck, Germany) using Quick ligation kit (New England Biolabs, USA) at the *EcoR*I and *Hind*III restriction site.

*EcoR*I and *Hind*III restriction sites are provided in the forward and reverse primers, respectively as underlined with bold characters. PCR was optimized in 50 µl reaction volume using GeneAmp PCR system 9700 (Applied Biosystems, USA) using high fidelity taq DNA polymerase (Bioline, UK). The PCR condition was optimized with initial denaturation at 94°C for 5 min; 35 cycles of amplification [a denaturation step at 94°C for 30 sec, annealing at 61°C for 45 sec, initial extension at 72°C for 2 min]; final extension at 72°C for 10 min. The PCR product was gel eluted and purified using GenElute gel extraction kit (Sigma Aldrich, USA).

The cloned AOx gene in pET28a (+) was transformed into competent BL21 (DE3) *E. coli* expression host by heat shock method [37]. Successful ligation into pET28a(+) and transformation into BL21 (DE3) was confirmed through fragment release after restriction digestion with *EcoR*I and *Hind*III restriction enzymes. Ligation of AOx gene at *EcoR*I and *Hind*III site allowed for Isopropyl β-D-1-thiogalactopyranoside (IPTG) inducible expression of apo-rAOx having both N- and C-terminal six-histidine tag for stringent affinity purification of this enzyme.

#### Expression, purification and *in-vitro* refolding of N'-HIS_6_-rAOx-HIS_6_-C' fusion protein from *E.coli*

Transformed *E.coli* BL21 (DE3) cells with pET28a-AOx recombinant plasmid were screened for positive clones and single colonies were grown overnight in Luria Bertani (LB) broth [1% (w/v) tryptone, 0.5% (w/v) yeast extract, 1% NaCl (w/v)] as primary culture supplemented with 50 µg ml^−1^ kanamycin antibiotic at 37°C and under constant shaking condition. The overnight grown primary culture was given a second passage in LB medium at a dilution of 1∶100 as a secondary culture and grown at the same condition specified above until the optical density (OD) at λ_660_ nm reaches mid-log phase (OD_660_ = 0.6–0.8) for induction with IPTG. To optimize small scale expression of apo-rAOx, the transformed secondary culture was induced with IPTG in concentration of 0, 0.25, 0.5 mM and a time point of 0, 4 and 8 h duration. Temperature of 15°C, 20°C, 25°C and 30°C were studied to optimize the induction temperature. Un-induced expression of apo-rAOX was also checked as a control.

Pure inclusion bodies containing the expressed protein were isolated by modifying the methodology of Schwanke *et al* [39]. The expression was scaled up in 1 liter culture volume. IPTG induction was carried out following the optimized parameters for small scale expression. Bacterial cells were harvested at 6000 x*g* at 4°C for 10 min. *E.coli* cells of approximately 2.4 g wet weight per liter of induced culture was used for inclusion body isolation and subsequent refolding studies. *E.coli* cells were lysed in lysis buffer (20 mM sodium phosphate buffer pH 7.4, 5 mM DTT, 1 mM PMSF) and the inclusion bodies were isolated by 12,000 x*g* for 30 min. The inclusion bodies were washed extensively using sodium deoxycholate (Sigma Aldrich, USA) at a concentration of 1% (w/v) in lysis buffer and kept at mild stirring condition for 1 h at RT and thereafter sonicated briefly (6–7 min, 15 sec on and 10 sec off, 30% amplitude). The inclusion bodies were pelleted at 12,000 x*g* for 30 min and resuspended in lysis buffer containing 2 M urea at a final total protein concentration of ∼2 mg ml^−1^. The pH of the solution was adjusted to 12.0 with 1 M NaOH and the solution was stirred at RT for 30 min. The pH was then reduced to 8.0 with 1 N acetic acid and centrifuged at 12,000 x*g* for 30 min. The supernatant containing the solubilized rAOx was collected and dialyzed overnight at 4°C against 20 mM sodium phosphate buffer pH 7.4 supplemented with 5% (v/v) glycerol.

After dialysis the solubilized protein mixture was applied onto Nickel-affinity pre-packed column (HisTrap Nickel Sepharose 6 Fast Flow crude, GE Healthcare, Sweden), pre-equilibrated with binding buffer (20 mM sodium phosphate buffer pH 7.4, 500 mM NaCl, 4 M urea and 20 mM immidazole). Column was washed with binding buffer prior to protein elution with elution buffer (20 mM sodium phosphate buffer pH 7.4, 500 mM NaCl, 4 M urea and 500 mM immidazole). SDS-PAGE of the eluted fractions was performed at 12% acrylamide concentration following standard protocol [24]. The fractions containing the protein of interest were pooled together and dialyzed overnight against 20 mM Tris-HCl buffer, pH 9.0 supplemented with 5% (v/v) glycerol, 5 mM DTT and 1 mM PMSF.

*In-vitro* activation and refolding of purified apo-rAOx with its co-factor FAD were performed following the protocol of Ruiz-Dueñas *et al* [35]. Briefly, an optimized protein concentration of ∼10 µg ml^−1^ of purified apo-rAOx was used in a refolding mixture comprising of 20 mM Tris-HCl buffer, pH 9.0 containing 2.5 mM glutathione oxidized (GSSG), 1 mM DTT, 35% glycerol, 0.3 M urea and 0.08 mM FAD. The refolding mixture was allowed to incubate at 16°C for a period of ∼80 h. A control, containing FAD and protein in 20 mM Tris-HCl buffer pH 9.0 (no DTT and GSSG) at similar experimental conditions was also studied for in-vitro folding efficiency.

#### Determination of enzyme activity and kinetics

Activity of rAOx from inclusion bodies was assayed using the following aryl alcohols (5 mM each): 3, 4-dimethoxybenzyl alcohol (veratryl alcohol), 3-methoxybenzyl alcohol (*m*-anisyl alcohol), 4-methoxybenzyl alcohol (*ρ*-anisyl alcohol) and benzyl alcohol. Temperature and pH optima were evaluated in 0.1 M sodium phosphate buffer, pH 6.0 [15], [35]. Enzyme activity was expressed in terms of units (U), where one unit is defined as that amount of enzyme that catalyzes the conversion of one micromole (1 µmol) of substrate into product in one minute under the conditions specified. Enzyme activities were calculated during the linear phase of oxidation of different aryl alcohols to their corresponding aldehydes using the molar absorbance of 3, 4-dimethoxybenzaldehyde (ε_310_ = 9300 M^−1^ cm^−1^), 3-methoxybenzaldehyde (ε_314_ = 2540 M^−1^ cm^−1^), 4-methoxybenzaldehyde (ε_285_ = 16,980 M^−1^ cm^−1^) and benzaldehyde (ε_250_ = 13,800 M^−1^ cm^−1^) (Guillén *et al*., 1992). Apparent *K_m_* and *k_cat_* values were estimated using Lineweaver–Burk plots.

#### Western blot analysis

The purified rAOX was blotted onto PVDF membrane and detected using mouse monoclonal anti-poly-histidine antibody and anti-mouse immunoglobulin (Fab specific)-peroxidase antibody (Sigma Aldrich, USA) as the primary and secondary antibody, respectively. The membrane was then developed with peroxidase substrate 3, 3′-diaminobenzidine (DAB) tetrahydrochloride hydrate (4 mg per 10 ml in PBS buffer) and spiked with 30% hydrogen peroxide.

#### MALDI-TOF mass spectrometry study of rAOx

Purified histidine-tagged protein was run in 12% SDS-PAGE. The gel was then stained with mass spectrometry compatible coomasie G-250 protocol of Candiano *et al* [4]. Target protein band was excised using a sterile scalpel. In gel tryptic digestion of protein was performed following trypsin profile in-gel digestion (IGD) kit (Sigma Aldrich, USA) following the manufacturer's protocol for coomasie stained gels. The processed sample from above step was mixed with MALDI compatible α-Cyano-4-hydroxycinnamic acid matrix (Sigma Aldrich, USA) in protein sample volume to matrix ratio of 1∶1. Sample protein for MALDI TOF/TOF was prepared and processed as stated briefly in previous section. Sample parent ions generated in MS were selected for further MS/MS analysis for better sequence coverage. The MS/MS database search was performed by ProteinPilot software (AB SCIEX, USA).

#### Determination of isoelectric point (p*I*) of rAOx

The theoritical p*I* was computed using online Compute p*I*/Mw tool (ExPASY Bioinformatics Resource Portal, Switzerland) and confirmed experimentally through 2D electrophoresis following the protocol of Gőrg *et al* [11]. Immobiline dry pH gradient strip (linear pH gradient 3–10, 7 cm, GE Healthcare, Sweden) was rehydrated overnight with rehydration solution supplemented with IPG buffer or Pharmalyte. Sample protein with rehydration buffer was reduced and alkylated. Sample was then actively rehydrated onto immobiline dry pH gradient strips by following the cup loading procedure described in manufacturer's instruction manual (GE Healthcare, Sweden). Focusing of IPG strip was done at 20°C and 50 µA current per strip. Running condition was optimized at 500 V for 1 h (step and hold) followed by 1000 V for 30 min (gradient), 5000 V for 1 h 30 min (gradient), finally ending with 5000 V for 30 min (step and hold). Focused strip was equilibrated in two steps for reduction and alkylation following standard kit's procedure described previously. The IPG strip was run in second dimension in 12% resolving gel along with standard protein molecular weight marker as reference. Gel was sealed with bromophenol-agarose sealing solution. Silver staining was performed by following the protocol of Chevallet *et al* [5] and the gel was scanned using ImageScanner III (GE Healthcare, Sweden). 2D gel spot was analysed manually.

Isoelectric point of the purified protein was studied using Zetasizer Nano series (Malvern Instruments Limited, UK) to reconfirm and validate the p*I* determined by using 2-D gel electrophoresis. Purified AOx in different pH adjusted 20 mM sodium phosphate buffer was prepared (pH 5.7 to pH 7.2 at a pH increment of 0.1) and was filtered through 0.22 micron syringe filter to remove dust particles. The filtered protein in different pH buffer was charged into specially designed folded capillary cell with gold plated beryllium/copper electrode (Malvern Instruments Limited, UK). Thus, a point where the plot of zeta potential versus pH gradient passes through zero zeta potential, re-confirmed the p*I* of the rAOx.

#### Fluorimetric identification of free FAD and rAOx-FAD holoenzyme in refolding buffer

Successful incorporation of cofactor FAD to apo-rAOx was studied through fluorescence excitation of free FAD (control with rAOx and FAD but no GSSG and DTT) and rAOx-FAD holoenzyme (after ∼80 h incubation and removing unbound free FAD) in refolding buffer at λ_443_ nm and monitoring the emission maxima at λ_527_ nm (FluoroMax 4, Horiba Scientific, Japan). A characteristic lowering of fluorescence intensity of refolded rAOx-FAD holoenzyme in comparison to free FAD suggests efficient incorporation of FAD into protein matrix.

#### Circular dichroism (CD) spectroscopy study

Conformational stability of rAOx was studied in different pH buffer environment ranging from pH 4.0 to pH 9.0. The stability of the protein secondary structure in both acidic and basic buffer conditions were evaluated through CD spectra acquired with JASCO-815 spectrometer (Jasco, Japan) equipped with circulating water bath at 25°C. Different buffer compositions at 20 mM salt concentration were prepared on the basis of its buffering capacity. Buffer with pH 4.0 and pH 5.0 were sodium acetate; pH 6.0 and pH 7.0 were sodium phosphate; pH 8.0 and pH 9.0 were Tris/HCl. The unbound FAD was removed from the bound refolded rAOx fraction by micro centrifugal filter units (Vectaspin, Sigma Aldrich, USA) having 20 kDa cut off size, prior to diluting the protein at a concentration of 50 µg ml^−1^ in 20 mM of respective buffer composition at different pH ranging from 9.0 to 4.0. A control of unfolded rAOx in refolding buffer devoid of GSSG and DTT were also studied similarly as above. Samples were placed in 0.1 cm (optical path length) cell. Spectra was recorded under constant nitrogen gas purging at a flow rate of 5 L min^−1^ from λ_190_ nm to λ_260_ nm with a resolution of 0.1 nm and a band width of 1.0 nm. Each spectrum was the average of two accumulations at a scanning speed of 20 nm min^−1^. Background spectra of each corresponding pH buffer of same molar concentration as in the sample were recorded under identical experimental conditions. Background spectra were subtracted from its corresponding pH sample spectrum. To estimate the content of secondary structure, CD spectra were analyzed using online CD spectra analysis program K2D [1] in the range from λ_200_ nm to λ_240_ nm available online in DichroWeb [47], [48].

#### Dynamic light scattering (DLS) analysis of protein

For DLS analysis, purified apo-rAOx eluted from Nickel affinity column was concentrated immediately using Vectaspin 20 centrifuge filters (Whatman, GE healthcare, UK). A final protein concentration of ∼10 µg ml^−1^ in refolding buffer with FAD (composition as mentioned above) was maintained in the study. Sample protein solution was incubated for ∼96 h duration at 16°C and after every 24 h sample protein was monitored for particle size distribution. Commercial wild type AOx (cAOX) from *Pichia pastoris* (Sigma Aldrich, USA) and bovine serum albumin (Sigma Aldrich, USA) were studied as a positive and negative control in an identical experimental condition and concentration.

## Results

### Peptide mass fingerprinting (pmf) analysis of microsomal protein extracts

**Figure 2:**
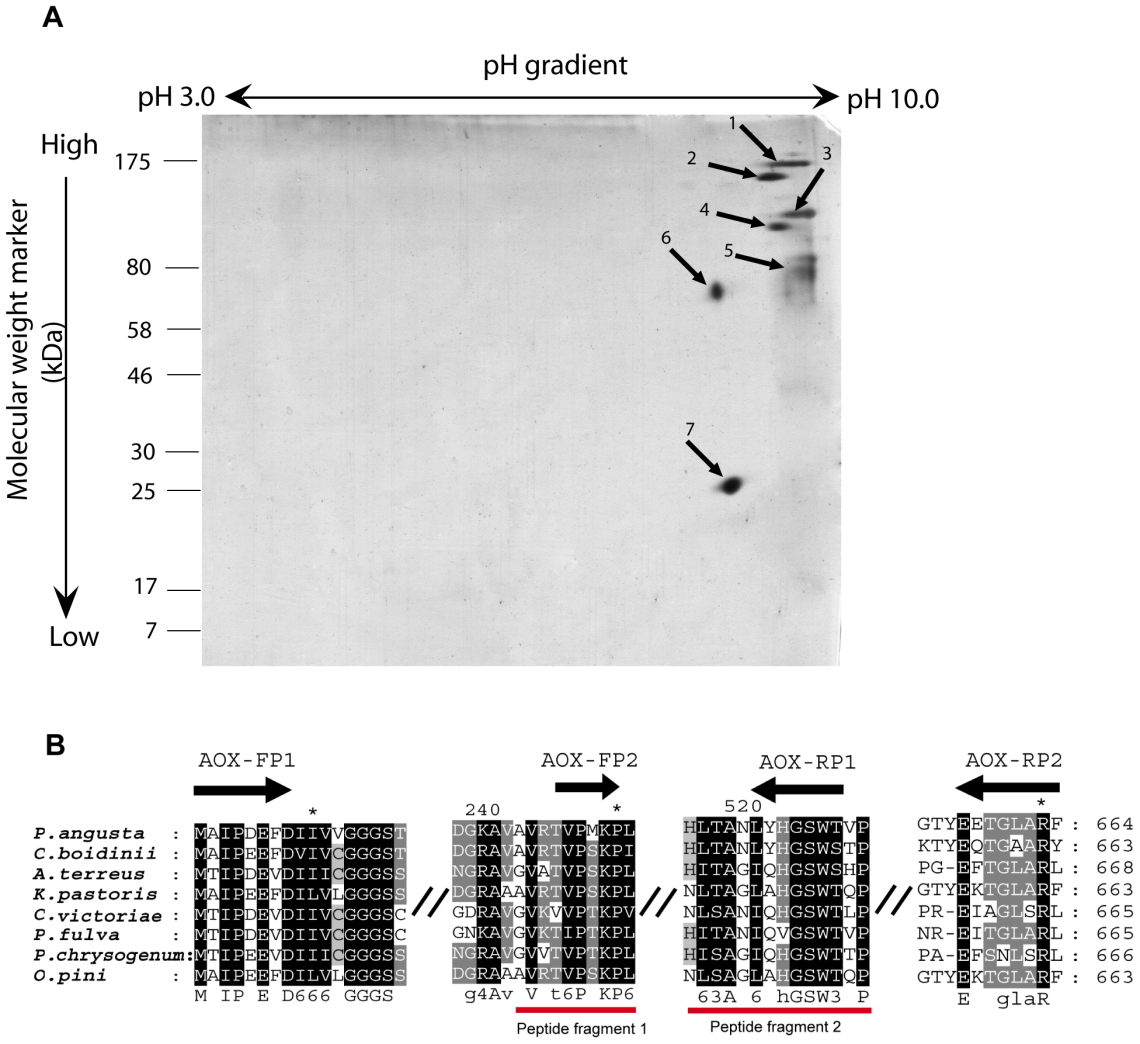
2D electrophoresis of microsomal membrane bound proteins and multiple sequence alignment of highly similar AOx proteins from filamentous fungi and yeast species showing the primers for overlapping PCR based on internal peptide fragments identified from pmf data of spot 6. (**A**). Mass spectrometry compatible silver stained 2D gel image of *A.terreus* MTCC6324 microsomal proteome. 150 µg of *A.terreus* microsomal protein having high AOx activity were resolved on 3–10 linear immobiline dry pH gradient strip as first dimension and subsequently run on 12% polyacrylamide resolving gel as second dimension. Seven spots (marked 1–7 along with arrow heads) showing the highest normalized volumes were analysed using MALDI-TOF-MS. (**B**). Internal peptide fragment 1 (GVATVPSKP) and fragment 2(NHITAGIQHGWSHP) shown in red bars, served as templates for designing overlapping PCR primers mapped as AOX-FP2 and AOX-RP1, respectively as an approach for characterizing full length AOx coding region. The gene identification numbers (GI) for the aligned amino acid sequences of AOxs are as follows: *P.angusta* (GI:113652); C.boidinii (GI:231528); *A.terreus* NIH2624 (GI:115437438); *K.pastoris* (GI: 2104963); *C. Victoriae* (GI: 13182929); *P.fulva* (GI: 9082281); *P.chrysogenum* (GI: 18028450). Sequences were aligned using CLUSTALW2 and viewed using GeneDock software. Forward and reverse primers are shown as black arrows with primer names mentioned above. Symbol (**//**) represents discontinuity in multiple sequence alignment. Highly conserved amino acid blocks are shaded in black.

About 150 µg of *A.terreus* MTCC6324 microsomal protein extracts containing AOx activity were resolved in 2D gel (**Fig. 2A**). A total of seven abundant spots detected in the gel were subjected to pmf studies. The m/z peaks produced from the spots were manually uploaded on FindPept tool. Hypothetical un-reviewed AOx protein sequence from *A.terreus* NIH2624 (GI: 115437438) available in National Center for Biotechnology Information (NCBI) was used as an input parameter to generate theoretical peptide masses for comparison with those obtained from the pmf peak list. Among the pmf spectra the one generated from spot six produced m/z peak corresponding to the target protein. Two conserved internal peptide sequences (peptide 1 and peptide 2) which were matched with the input AOx sequence (**Table 1**) were selected. The sequence information of the peptide fragments 1 and 2 were utilized to design forward primer (AOx-FP2) for second overlapping PCR and reverse primer (AOx-RP1) for first PCR, respectively (**Fig. 2B**).

**Table 1:** Matching peptides of AOx corresponding to conserved internal amino acid sequence as predicted by FindPept pmf search for tryptic digest of 2-D gel spot 6.

User mass	DB mass	Δmass (daltons)	Peptide	Position
855.074	855.493	0.419	Peptide 1: GVATVPSKP	239–247
1641.917	1641.788	−0.128	Peptide 2: NHITAGIQHGSWSHP	511–525

The pmf peak list (m/z values) generated from spot 6 of 2D gel was manually uploaded on FindPept online tool. Detectable m/z values were matched with the theoretical peptide masses generated from the virtual tryptic digest of query amino acid sequence of AOx from *A.terreus* NIH2624 strain as input. The output result shows user mass (practical), database mass (theoretical), delta mass (theoretical minus practical mass), peptide sequence and corresponding position in query sequence. The above table highlights only those two peptide fragments which correspond to conserved internal amino acid blocks from multiple sequence alignment of similar AOx from other filamentous fungi and yeast species.

### Gene amplification and construction of full length AOx

**Figure 3:**
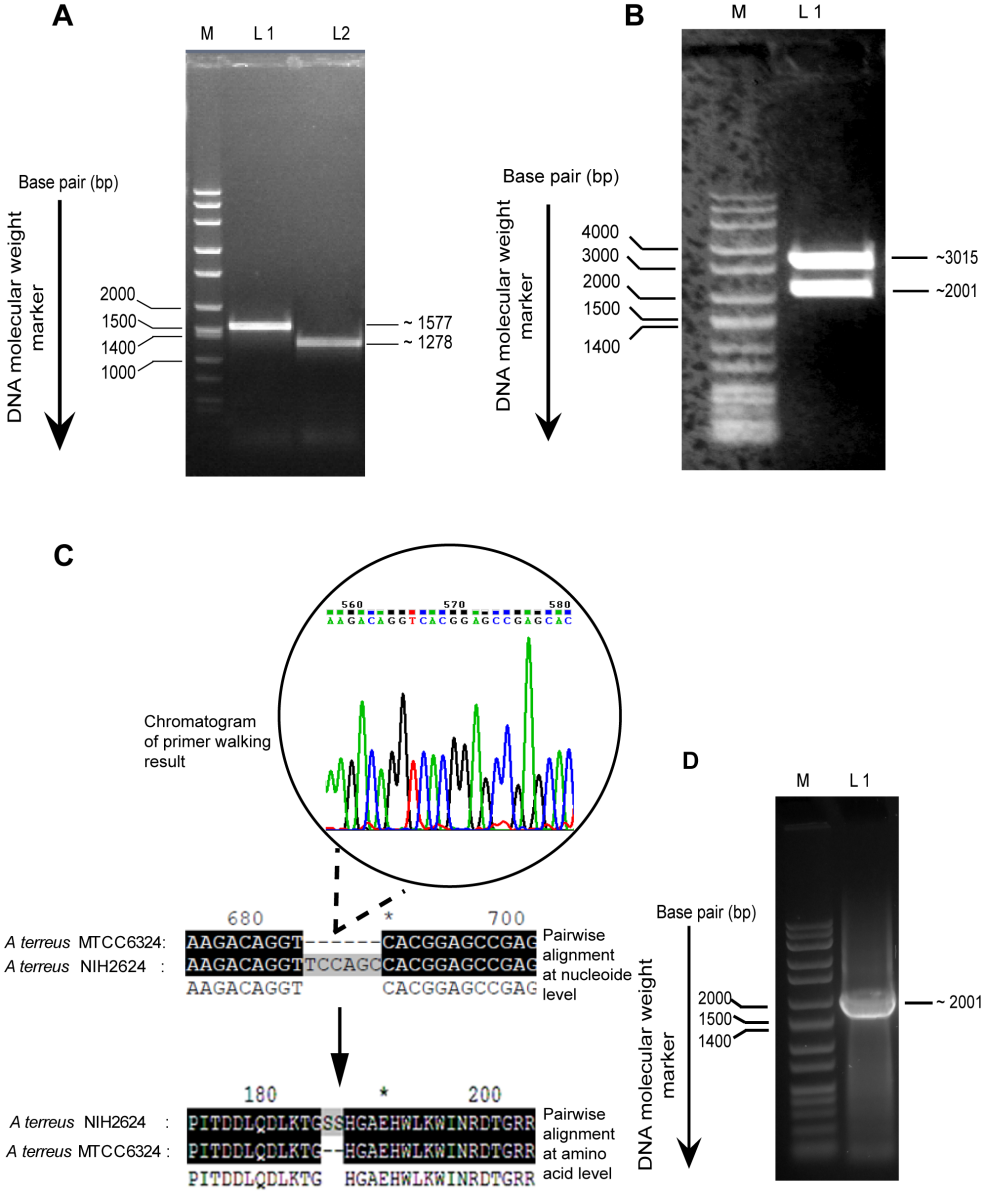
Ligation of overlapping PCR products at common restriction site and the full length AOx cDNA clone confirmation in TA vector through restriction digestion and double stranded DNA sequencing by primer walking. (**A**). Amplified overlapping PCR amplicons using *A.terreus* MTCC 6324 cDNA as PCR template. Lane M represents wide range DNA marker, lane L1 represents PCR 1, a ∼1577 bp cDNA fragment of AOx from its start codon, lane L2 represents PCR 2, a ∼1278 bp overlapping cDNA fragment of AOx till the stop codon. Qualitative gel analysis was performed in 0.8% agarose concentration stained with ethidium bromide. (**B**). Restriction digestion pattern of ligated overlapping PCR fragments in TA vector with *EcoR*I restriction enzyme. The release of ∼2001 bp full length AOx fragment from TA vector backbone of ∼3015 bp was evident. Qualitative gel analysis was performed in 0.8% agarose concentration stained with ethidium bromide. (**C**). Pair-wise sequence alignment of primer walking DNA sequence with the un-reviewed AOx sequence information from *A.terreus* NIH strain at nucleotide and amino acid level shows a deletion of six contiguous nucleotide sequences (position 685–690) which codes for two serine residues at position 186 and 187. Corresponding sharp nucleotide chromatogram of the deleted region is highlighted, confirming the good quality of the sequencing data and ruling out any possible error in sequencing. Highly similar residues are highlighted in black. (**D**). Qualitative agarose gel for PCR amplification of full length AOx from TA vector. Lane M is a wide range DNA marker, lane L1, shows PCR amplicon of AOx at ∼20001 bp using forward and reverse primers with *EcoR*I and *Hind*III restriction sites, respectively.

**Figure 4:**
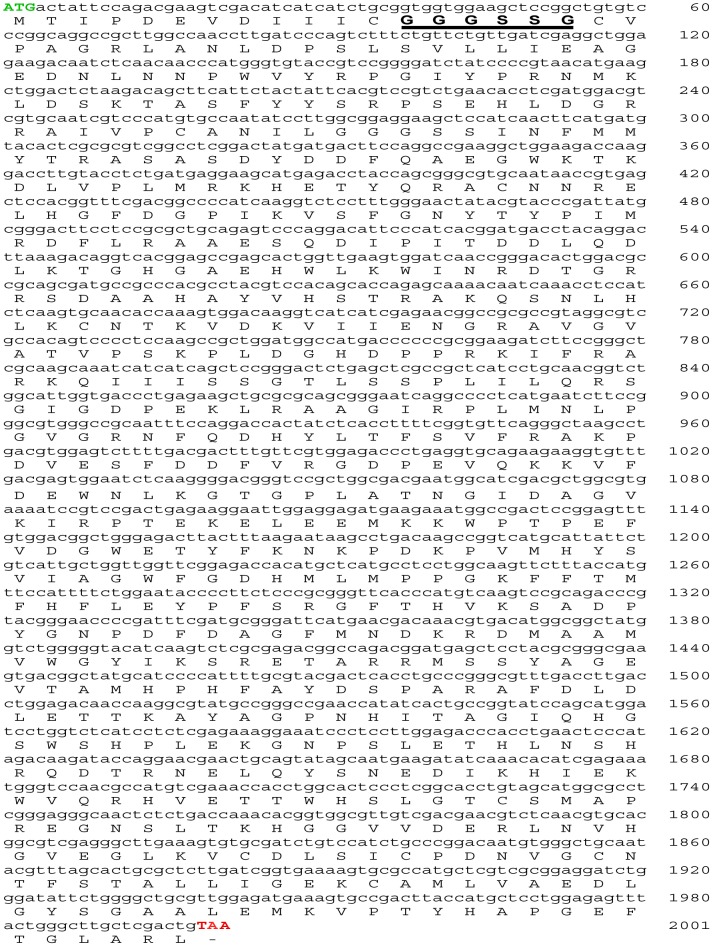
Nucleotide and deduced amino acid sequence of AOx from *A.terreus* MTCC6324. Double stranded primer walking confirmed an ORF of 2001(−) denotes a stop codon. The N-terminal conserved amino acids taking part in Rossmann fold architecture (GXGXXG motif) are underlined in black with its residues in bold. The full length cDNA is submitted to NCBI GenBank with accession no: JX139751.

The cDNA of AOx was constructed from total RNA isolated from the *n*-hexadecane induced *A.terreus* MTCC6324 cells by Reverse Transcriptase-Polymerase Chain Reaction (RT-PCR) mediated first strand synthesis using random hexamer primer probes followed by gene specific overlapping PCR to obtain full length coding region. The two PCR fragments, one (fragment 1) harboring the start codon till the end of reverse primer AOx-RP1 (PCR 1) and second (fragment 2), from the start of forward primer AOx-FP2 till the stop codon (PCR 2) proved successful (**Fig. 3A**) and DNA sequencing of individual PCR amplicon validated the nucleotide sequence read of ∼1577 bp and ∼1278 bp, respectively having 98% sequence homology with NIH *AOx* sequence. Restriction digestion analysis with *Nde*I and *Bgl*II confirmed the orientation of individual PCR fragments in TA cloning vector. The orientation of fragment 1 consisting of the *AOx* gene fragment from start codon was confirmed by the release of ∼840 bp and ∼3752 bp fragments, whereas the orientation of fragment 2 was confirmed by the release of ∼1278 bp and ∼3055 bp fragment (**Fig. S1**). The fragment 1 (∼3752 bp) with the start codon (ATG…) was ligated with fragment 2 (∼1278 bp) containing the stop codon (…TAA) to clone the full length coding sequence of *AOx* gene from *A.terreus* MTCC6324 in TA cloning vector. The insert in the clone was confirmed by digestion with *EcoR*I restriction enzyme, releasing an intact fragment at ∼2001 bp (**Fig. 3B**). Finally, a double stranded primer walking was performed to obtain the full length coding sequence information of AOx gene, confirming 2001 bp open reading frame. The sequence was analyzed for any probable mutations and its sequence similarity in NCBI database was adjudged with Basic Local Alignment Search Tool (BLAST). The sequence analysis revealed a novel AOx gene containing few mismatch and a deletion of six contiguous stretch of nucleotides from position 685 to 690 coding for two serine residues was prominent when pair wise aligned with un-reviewed conceptually translated sequence information of AOx from *A.terreus* NIH2624 strain available in NCBI GenBank (**Fig. 3C**). The AOx coding region was amplified using gene specific forward (AOX-pET28a-F) and reverse primers (AOX-pET28a-R) with *EcoR*I and *Hind*III restriction sites, respectively. A single PCR amplicon at ∼2000 bp was observed on agarose gel (**Fig. 3D**) and was subsequently ligated into *E.coli* expression vector pET28a (+) followed by transformation into expression host *E.coli* BL21 (DE3) for production of recombinant protein. The recombinant clones were selected and confirmed by double digestion with *EcoR*I and *Hind*III releasing a fragment of ∼2001 bp AOx clone (**Fig. S2**). The presence of same cDNA in correct open reading frame was confirmed through DNA sequencing and matched with our submitted sequence (GI: 398707520). The sequence is a novel AOx gene from *A.terreus* experimentally validated through wet lab studies as described below and the sequence data was submitted to the NCBI GenBank database under the accession number JX139751 (**Fig. 4**).

### Expression, purification and activation of rAOx

**Figure 5:**
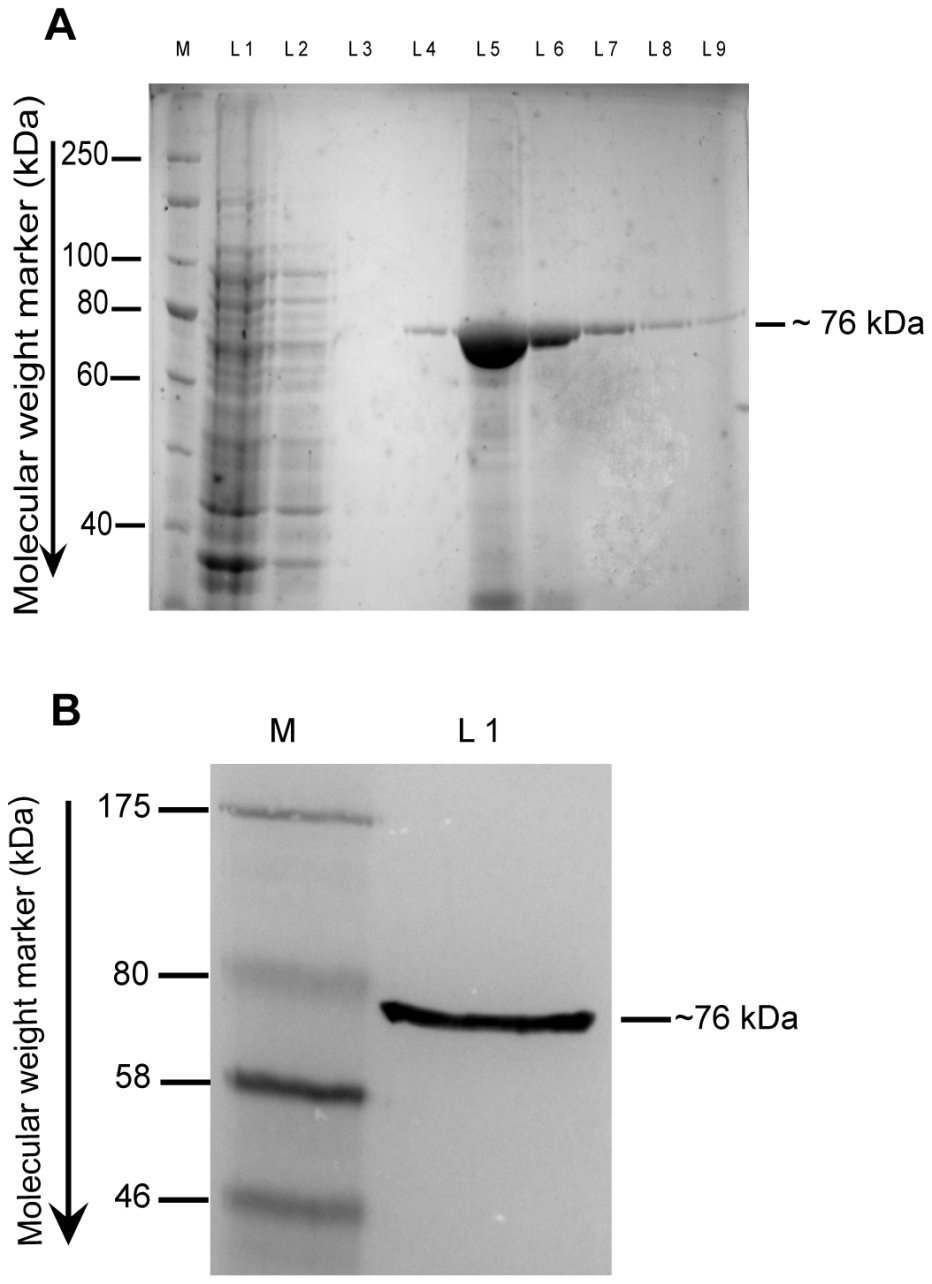
Purification profile of apo-rAOx and western blot analysis. (**A**). Purification profile of apo-rAOx using Nickel affinity chromatography. Lane M is protein molecular weight marker, lane L1 is crude solubilized supernatant, lane L2 is the unbound fraction from column, lane L3 is the washed flow through fraction, lane L4 –L9 are the elution fraction from column. Purified protein band at ∼76 kDa is shown with a black bar. (**B**). Western blot of the purified rAOx against N- and C-terminal 6× polyhistidine tags. Lane M is a protein molecular weight marker and lane L1 is the developed ∼76 kDa rAOx blot on PVDF membrane shown against a black bar.

A 2001 bp coding region of *AOx* was expressed in *E. coli* BL21 (DE3) strain by induction with 0.25 mM Isopropyl β-D-1-thiogalactopyranoside (IPTG) for 4 h (**Fig. S3**) at 28°C (**Fig. S4**). The ∼76 kDa apoenzyme rAOx (apo-rAOx) band appeared mainly in the insoluble fraction of the cell lysate. Approximately, 2.4 g wet cell of recombinant *E.coli* BL21 (DE3) was pelleted for isolation and purification of inclusion body after cell lysis as described in experimental section. Pure inclusion bodies were solubilized under basic pH environment to avoid any aggregation before purifying through single nickel affinity chromatography step. SDS-PAGE analysis of eluted fractions from HisTrap nickel sepharose 6 affinity column (**Fig. 5A**) showed that the purification protocol successfully yielded a solubilized homogenous apo-rAOx band at ∼76 kDa. A homogenous apo-rAOx protein yield of ∼10.2 mg of protein per 2.4 g wet cell weight per liter was achieved. Western blot analysis confirmed that the purified protein was expressed as a histidine tag at approximately the same molecular weight (∼76 kDa) as in the SDS PAGE gel (**Fig. 5B**).

*In-vitro* re-folding of apo-rAOx with its co-factor FAD was performed after the purification. In vitro folding was performed by using ∼10 µg ml^−1^ of purified apo-rAOx in the refolding buffer. Notably, *in-vitro* refolding of apo-rAOx using protein concentration above the optimized value (∼10 µg ml^−1^) was found to be highly aggregating and protein precipitation was evident in the solution, thereby hindering the refolding process. Thus, single step purification of rAOx with nickel affinity chromatography followed by *in-vitro* activation with co-factor FAD proved to be efficient.

### Biophysical and functional characterization of rAOx

Mass of apo-rAOx with his-tag measured by MALDI-TOF/TOF was 74,614 Da (**Fig. S5**) which was in good co-relation with the theoretical mass of 74475.26 Da calculated by using deduced amino acid sequences that void his-tag. Protein pilot database search of MS/MS data revealed a significant match with hypothetical AOx protein sequence from *A.terreus* NIH strain (GI: 115437438) with a score of 165 and sequence coverage of 28%.

The isoelectric point of the apo-rAOx (with his-tags) was studied by 2D electrophoresis and was verified with zeta potential studies (in pH range 5.7–7.2). In both independent studies conducted, the p*I* of the protein was confirmed to be 6.4 (**Fig. S6A**) and 6.52 (**Fig. S6B**), respectively. The experimental p*I* values were in good agreement with the theoretical p*I* of 6.81, taking into consideration the N- and C- terminal his-tags. (ExPASy Compute p*I*/Mw tool).

**Figure 6:**
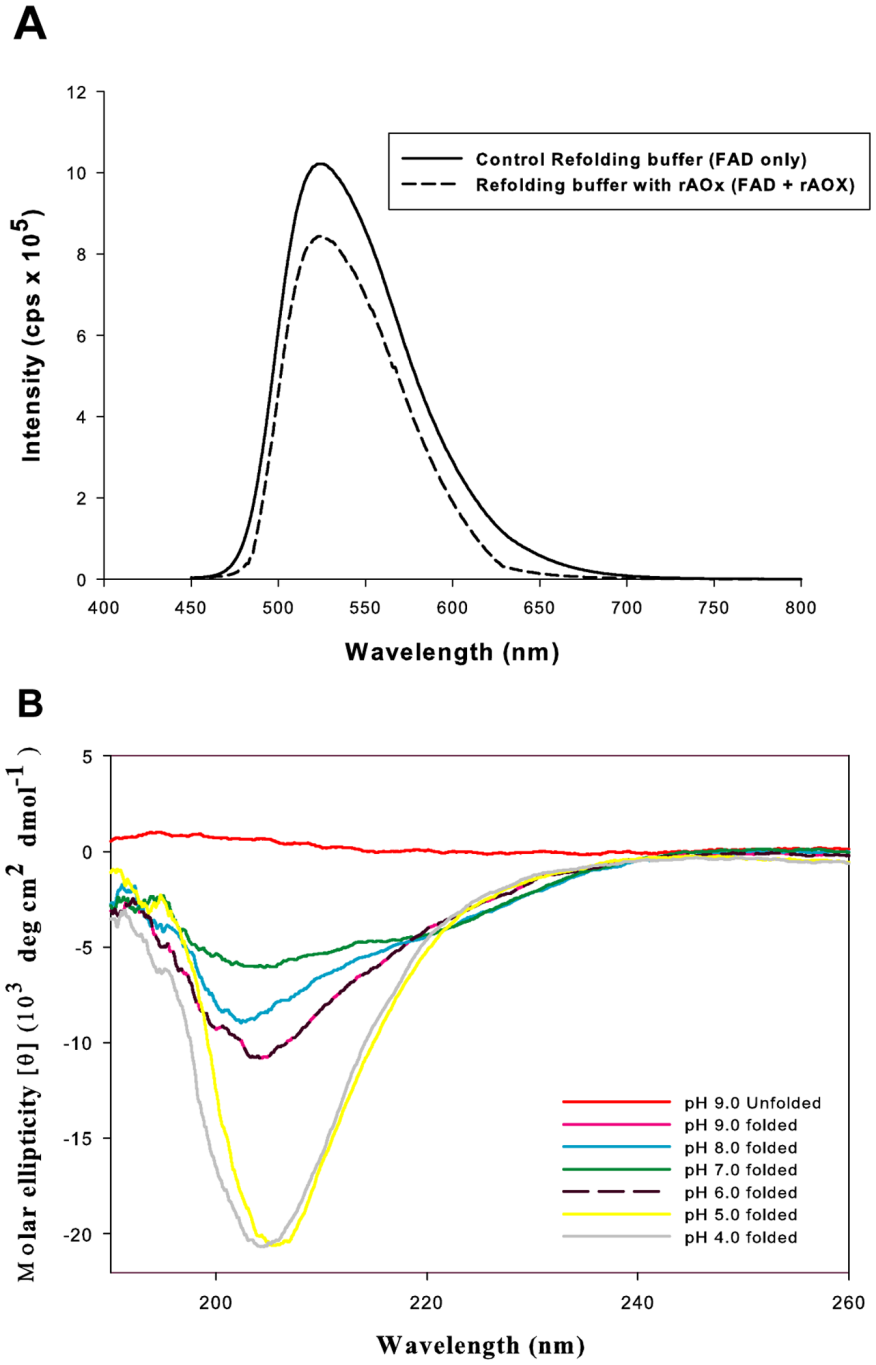
Fluorescence spectra and Circular dichroic spectra of refolded rAOx with co-factor FAD as holoenzyme. (A). Decrease in fluorescence emission maxima of free FAD (solid black curve) and FAD reconstituted holoenzyme (rAOx) (black dashed curve) at λ_∼527_ nm diluted in 20 mM sodium phosphate buffer pH 9.0 shows the quenching due to successful incorporation of FAD in the protein core during refolding after ∼80 h incubation at specified condition. (B). Dichroic spectra of rAOx from λ_190_ nm to λ_260_ nm in both basic and acidic environment taken after ∼80 h incubation at 4°C. Spectrum of purified apo-rAOx in identical folding buffer without glutathione and DTT was also studied as a control (red spectrum). Spectra of FAD bound rAOx fraction were recorded after removal of unbound FAD by micro-centrifugal filtration and diluting the holoenzyme rAOx in 20 mM of the respective pH buffer at a range from 9.0 to 4.0. CD spectra at pH 9.0 (pink spectrum) and pH 6.0 (black dashed spectrum) overlaps due to similar conformational stability at the respective pH environment.

Fluorescence emission spectra of FAD reconstituted apo-rAOx (rAOx) and free FAD are shown in **Fig. 6A**. Excitation at λ_443_ nm gave similar emission spectra for both the samples with emission peak at ∼λ_527_ nm. A lower fluorescence intensity of the holoenzyme relative to that of free FAD inferred efficient incorporation of the cofactor FAD in the apo-rAOx matrix during its refolding process. At the same protein concentration, the FAD fluorescence intensity of rAOx was about 80% to that of apo-AOx containing free FAD in presence of refolding buffer. This decrease in fluorescence of FAD upon coupling to apo-rAOx is likely to be caused by the surrounding protein shell.

CD spectra of rAOx were analyzed in different pH buffer environment from pH 9.0 to pH 4.0, after ∼80 h incubation at 16°C in refolding buffer after diluting it with appropriate pH buffer. CD spectra recorded from λ_190_ nm-λ_260_ nm (**Fig. 6B**) revealed a stable conformation of folded rAOx in the above mentioned pH range and was found to be an ordered protein with α-helix, β-strand and random/non-regular structures as 28±1%, 33±2% and 39±2%, respectively as predicted through CD spectra analysis program K2D. CD spectra at pH 9.0 and pH 6.0 are overlapping which reflects similar stable secondary structure composition at both the pH conditions. A control study of an unfolded protein in identical folding buffer without glutathione oxidize and DTT was carried out where the α-helix, β-strand and random/non-regular structures were 8±1%, 44±3% and 48±2%, respectively, where a high β-strand and random/non-regular structures are predominant, a characteristic of an unfolded protein.

**Figure 7:**
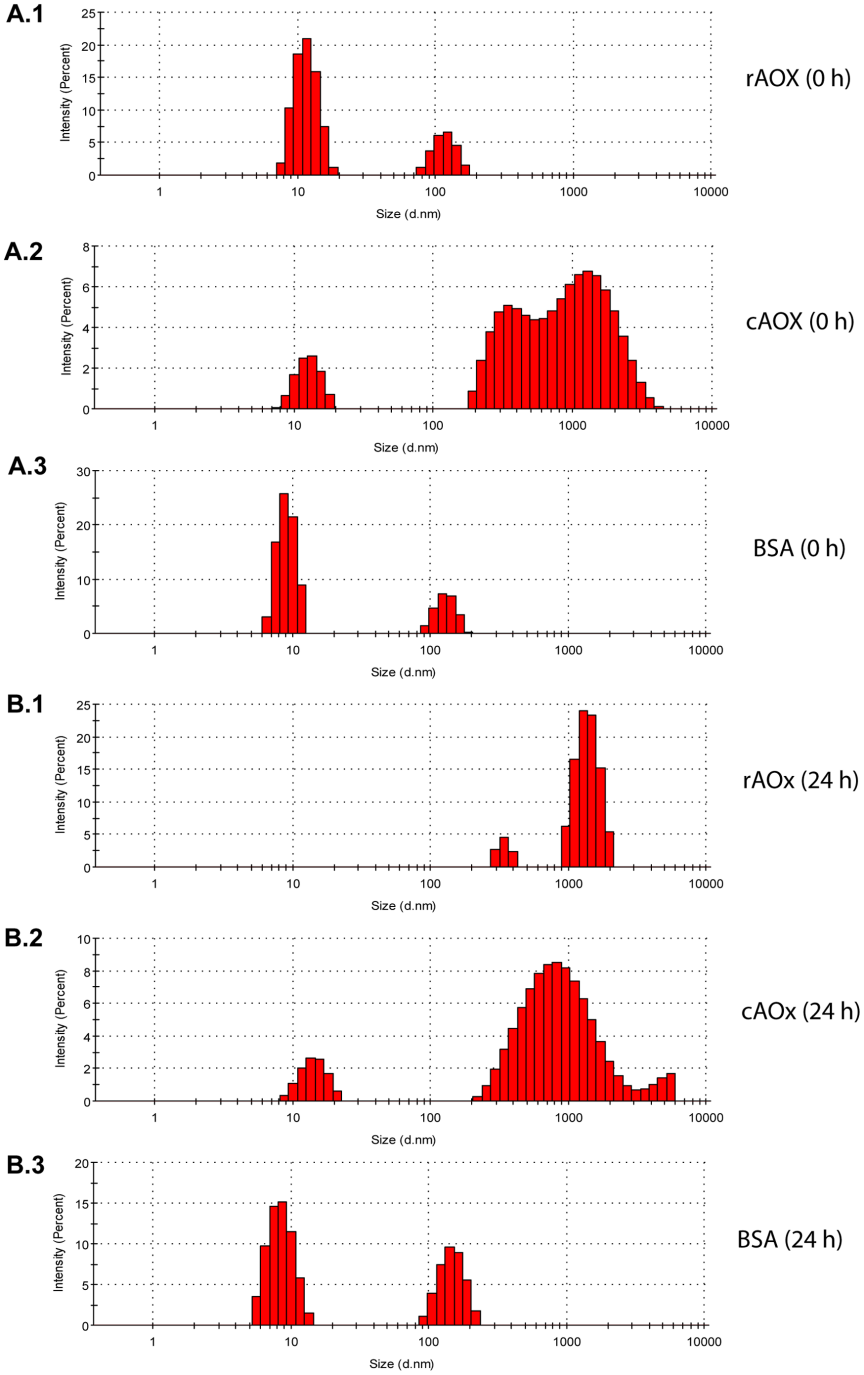
DLS analysis of rAOx, cAOx from *Pichia pastoris* and BSA for 0 h and 24 h incubation, respectively at 16°C. Readings were taken after removal of unbound FAD through micro-centrifugal filtration and subsequent filtering through 0.22 micron syringe filter of each time-point samples. (**A**). Onset of aggregation immediately after mixing the purified apo-rAOx with FAD in refolding buffer (0 h), the presence of peak at diameter (d) = ∼10 nm (panel **A1**), confirms the onset of aggregation. Panel **A2** shows the 0 h DLS signal of cAOx containing high aggregation reflected by a broad peak at diameter (d) = ∼1000 nm. Panel **A3** represents the aggregation profile of BSA at 0 h. (**B**). Panel **B1**, shows the highly aggregated complex formed after 24 h incubation of rAOx with major peak shift to diameter (d) = ∼1000 nm. Panel **B2** shows the constant aggregation profile of cAOx with no major peak shift when compared to 0 h data. Panel **B3** represents the constant aggregation profile of BSA, no major peak shift suggests no higher aggregated complex formed after incubation for 24 h and acted as a positive control.

DLS studies with rAOx confirmed the highly aggregating tendency of the protein due to intermolecular interactions at high protein concentration. Purified apo-rAOx protein at a concentration of ∼10 µg ml^−1^ (same concentration as in refolding studies) was monitored for 96 h duration in refolding condition with FAD. Simultaneously, we have checked the aggregation profile of a commercial AOx (cAOx) from *pichia pastoris* and bovine serum albumin (BSA) as a positive and negative control, respectively. At 0 h, rAOx exhibited aggregation tendency with a major signal at ∼10 nm diameter, indicating the onset of aggregation (**Fig. 7A1**). Within 24 h of incubation the major signal drastically shifted to ∼1000 nm diameter, suggesting high aggregation pattern (**Fig. 7B1**). Further incubation for 48 h, 72 h and 96 h showed a constant major peak at ∼1000 nm diameter (**data not shown**). In our positive control the cAOx showed high aggregation pattern at 0 h (**Fig. 7A2**) which was consistent over a period of 24 h (**Fig. 7B2**). Further incubation for 48 h, 72 h and 96 h showed constant peak (**data not shown**). Aggregation profile of BSA was similarly studied as a negative control to rule out the possibility of aggregation due to refolding buffer components and no significant aggregation pattern was observed (**Fig. 7A3** and **Fig. 7B3**). The aggregation study clearly demonstrates the intrinsic tendency of apo-rAOx to form high aggregates and thus posing a major challenge in hindering the efficient *in-vitro* refolding with FAD.

**Figure 8:**
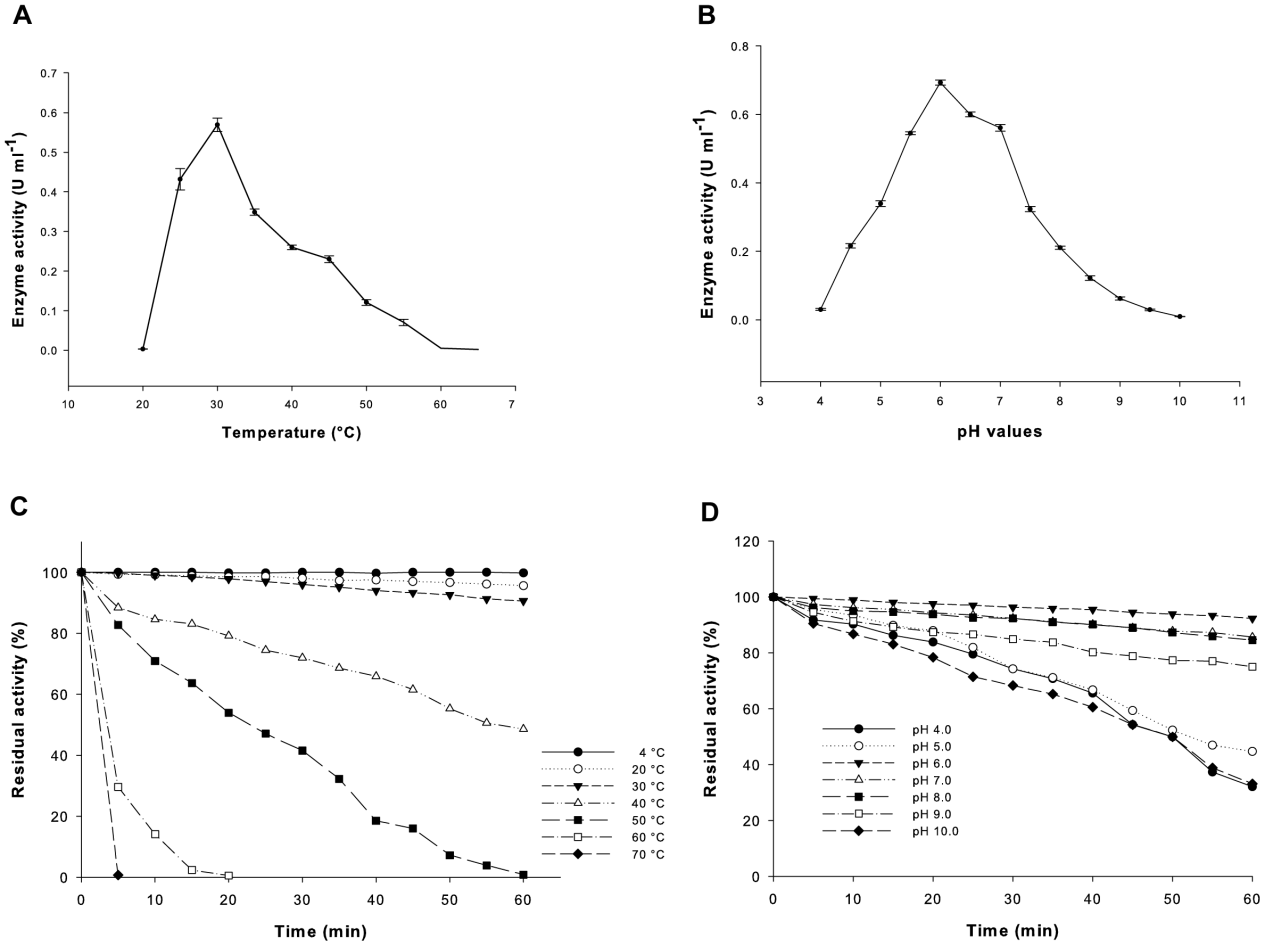
Studies on temperature, pH optima, thermal and pH stability of rAOx. Effect of temperature (**A**) and pH (**B**) on the activity of rAOx was studied using 5 mM *ρ*-anisyl alcohol in 100 mM sodium phosphate buffer. Data are the mean of three (n = 3) independent experiments. (**C**) Thermal stability of rAOx when incubated for 1 h at different termperature. Residual activity (%) was calculated taking enzyme activity value (in U mg^−1^) with 5 mM *ρ*-anisyl alcohol as substrate in 100 mM sodium phosphate buffer, pH 6.0 as 100%. (**D**) pH stability of rAOx at different pH buffer monitored for 1 h. Residual activity was calculated as mentioned above.

*In-vitro* refolded rAOx showed an optimum temperature and pH of 30°C and pH 6.0, respectively for its activity (**Fig. 8A** and **8B**). Thermal stability of activated rAOx showed that the enzyme was stable upto 30°C and the stability was greatly reduced at 40°C (50% inactivation) and lead to 100% inactivation after 5 min at 70°C (**Fig. 8C**). Moreover the rAOx demonstrated partial pH stability in range from pH 6.0 to pH 9.0 after 1 h (**Fig. 8D**).

### Molecular modeling and docking studies of rAOx

**Figure 9:**
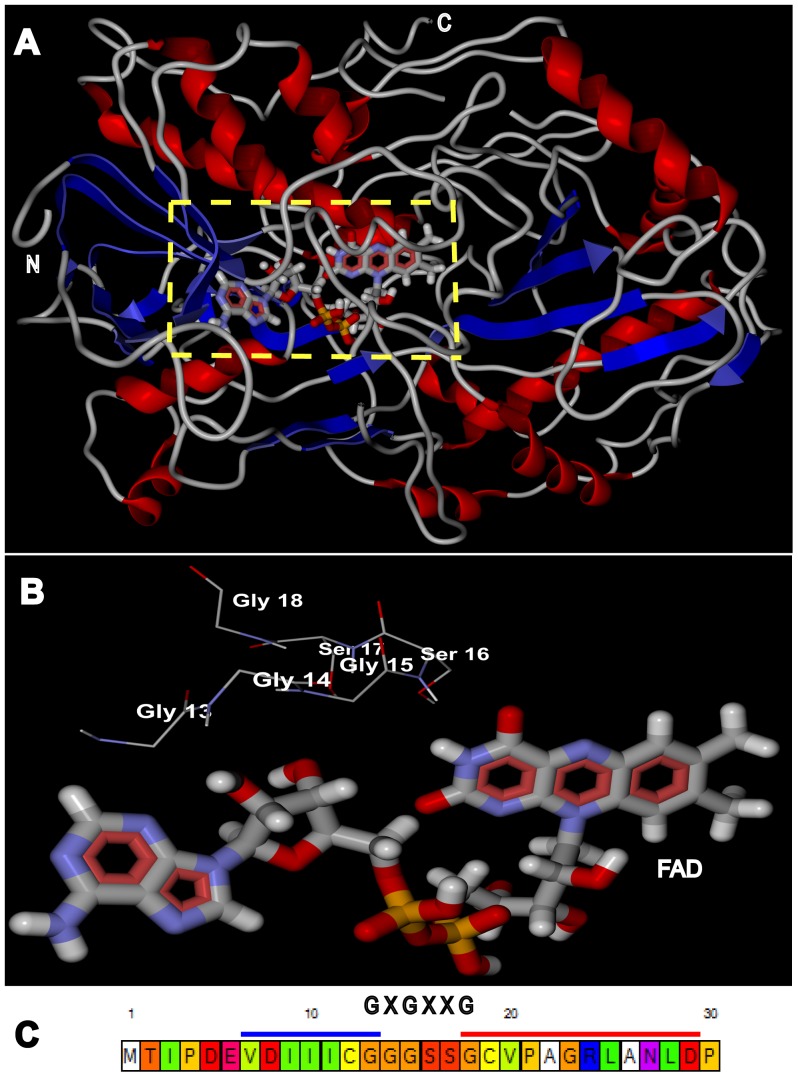
I-TASSER predicted *ab-initio* model of apo-rAOx docked with its co-factor FAD. (**A**). The best model (predicted by I-TASSER) docked with FAD using Molegro Virtual Docker. FAD is represented as Corey-Pauling-Koltun (CPK) model bound at its conserved Rossmann fold motif (GXGXXG) highlighted by yellow dotted square with its residue labeled as shown as a magnified view in panel (**B**) in the model and also shown against the N-terminal loops region present between the first β-sheet (highlighted as blue bar) and first α-helix (highlighted as red bar) in the lower panel (**C**). The loop region takes part in non-covalent interaction with FAD, thus stabilizing the overall structure. Red ribbon shows the α-helices, blue ribbon depicts β-sheets and loops are shown in grey tubular wire.

Ab-initio based modeling using I-TASSER server predicted a model of rAOx with a confidence score (C-score) of −0.92 and estimated accuracy (TM-score) of 0.60±0.14 (**Fig. 9A**). The stereo chemical quality of the predicted model was evaluated through PROCHECK producing a number of postscript plots among which the Ramchadran plot predicted 79.5% residues lying in the most favored region. The crystal structures used as templates in predicting our model were also assessed to validate the model. The selected model was found to be satisfactory for the calculated stereo-chemical parameters.

**Figure 10:**
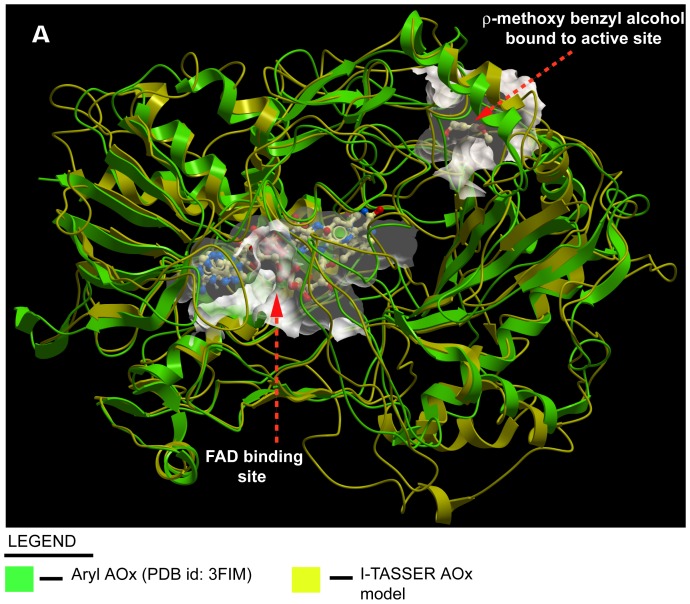
3D superimposition of predicted rAOx holoenzyme model with that of the holoenzyme aryl alcohol oxidase crystal structure (PDB id: 3FIM). The 3D superimposition of our predicted FAD bound rAOx model (shown in yellow color ribbon structure) with the chain B of crystal structure of holoenzyme AOx (shown in green color ribbon structure) from *P. eryngii* (PDB id: 3FIM) using Molsoft ICM browser (www.molsoft.com). Both the FAD molecules from respective protein models and the *ρ*-methoxybenzyl alcohol as the docked substrate molecule to the model rAOx from *A.terreus* MTCC6324 are shown as Corey-Pauling-Koltun (CPK) model with its individual binding pockets highlighted in white scaffolds.

The modeled apo-rAOx was successfully docked with its co-factor FAD and was found to be in consistent with the conserved FAD binding amino acid residues, GXGXXG motif (where × being any residue) as Rossmann fold reported previously [32] (**Fig. 9B** and **C**). In our control study, aryl-AOx crystal structure (pdb id: 3FIM) in apoenzyme form was docked simultaneously with our FAD best pose (judged by using MolDock scoring matrix) and were superimposed (**data not shown**). Superimposition of both the docked FAD molecules were analysed using Swiss PDB Viewer version 4.1 in ExPASy bioinformatics resource portal [14] and a rationale root-mean-square distance (rmsd) signified a satisfactory evaluation of our docking performance. 3D superimposition of our predicted model with aryl AOx crystal structure (pdb id: 3FIM) revealed significant homology with chain B of the structure (**Fig. 10**), although at their amino acid sequence level non-homology is predominant (**data not shown**).

**Figure 11:**
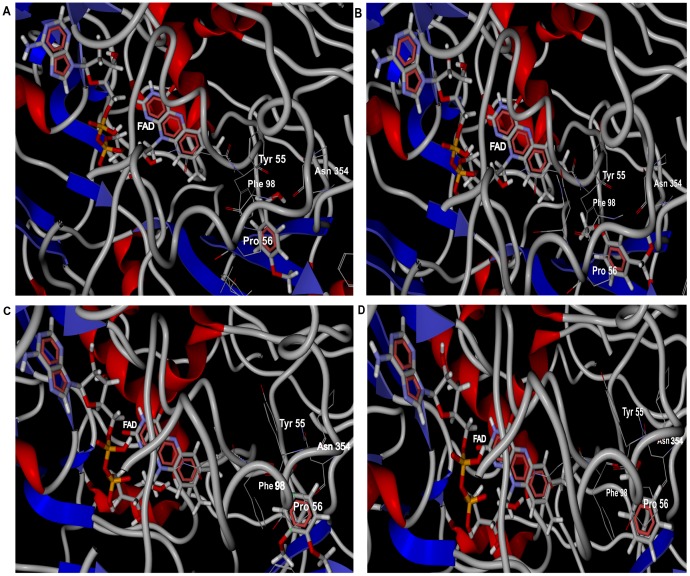
Docking view of modeled rAOx (FAD docked) with its alcohol substrates. Docking view of aromatic alcohols (highlighted as thick stick CPK model) as substrates with FAD docked (highlighted as thick stick CPK model) apo-rAOx holoenzyme complex. Conserved amino acid residues hypothesized to take part in catalytic reaction in oxidizing its substrates are highlighted as thin stick Corey-Pauling-Koltun (CPK) model with its residues labelled. Panel (**A**), (**B**), (**C**) and (**D**) shows the close-up docking view generated by Molegro Virtual Docker version 4.0.2 (CLC bio-Qiagen company) of *ρ*-methoxybenzyl alcohol; *m*-methoxybenzyl alcohol; 3,4 dimethoxybenzyl alcohol and benzyl alcohol, respectively.

Flexible docking simulation with reported aromatic alcohols with FAD bound modeled rAOx was performed and the best pose of each ligand (aryl-alcohols) were assessed based on MolDock score (**Fig. 11A, B, C, D**). The results clearly demonstrates the substrate affinity for our predicted AOx model for aromatic alcohols in order of preference with its total binding energy in parenthesis as 4-methoxybenzyl alcohol (−82.87 kJ mol^−1^) >3-methoxybenzyl alcohol (−74.12 kJ mol^−1^)>3, 4 dimethoxybenzyl alcohol (−71.75 kJ mol^−1^)> benzyl alcohol (−56.45 kJ mol^−1^).

Presence of amino acid residues Tyr 55, Pro 56, Phe 98 and Asn 354 were found to be conserved near the substrate binding site (**Fig. S7**). The presence of aromatic amino acid Tyr 55 and Phe 98 was hypothesized to contribute in cation-*π* interaction, an important signature pattern observed and reported for other aryl alcohol oxidase [16], [17].

### Enzyme activity and kinetic studies of AOx

A range of alcohol substrates for activity of the rAOx was studied and found that only the aryl alcohol substrates showed detectable activity for the recombinant enzyme. Steady-state kinetic parameters of rAOx were evaluated based on its catalytic activity exhibited for the oxidation of four different aromatic alcohols. Kinetic constants pertaining to *K_m_*, *k_cat_* and *k_ca_*_t_/*K_m_* for each of the substrates tested revealed benzyl alcohol as a poor substrate while 4-methoxybenzyl alcohol (*ρ*-anisyl alcohol) being the best aromatic alcohol substrate followed by 3-methoxybenzyl alcohol (*m*-anisyl alcohol) and 3, 4-dimethoxybenzyl alcohol (veratryl alcohol) (**Table 2**). Catalytic efficiency as predicted by *k_cat_*/*K_m_* for 4-methoxybenzyl alcohol was 7829.5 min^−1^ mM^−1^, which was the highest among the four aromatic alcohols tested.

**Table 2:** Steady-state kinetic parameters of *in-vitro* refolded recombinant alcohol oxidase from *E.coli*.

Aryl alcohol substrates	rAOx from *E. coli*			
	*K*_m_ (mM)	Specific activity (U mg^−1^)	*k*_cat_ (min^−1^)	*k*_cat_/*K*_m_ (min^−1^ mM^−1^)
*ρ*-anisyl alcohol	1.08	111.11	8455.86	7829.50
*m*-anisyl alcohol	2.88	83.33	6341.70	2201.98
Veratryl alcohol	4.91	91.00	6925.42	1410.47
Benzyl alcohol	10.80	1.70	129.38	11.98

Mean *K*_m_, *k*_cat_ and *k*_cat_/*K*_m_ values were determined and all assays were performed in replicates of 3 (n = 3).

## Discussion

Characterization of AOx genes from methylotropic yeasts has been studied extensively, while the same is yet to be performed adequately in case of filamentous fungi [12]. Among the fungi, few basidiomycetes have been studied deeply on the function and molecular characteristics of the AOx produced by these strains. The function of the AOx from these sources is mostly ascribed to the oxidation of aryl alcohols to produce H_2_O_2_, which is involved in the peroxidative degradation of lignin [36]. Moreover, these aryl AOxs are produced extra cellularly by these lignin-degrading fungi and aryl alcohols are the known inducers for them. Conversely, *A.terreus* produces AOx intracellularly along with cytochrome P450 (CYP450) in the microsomes of the cell and the enzyme is well induced by the non-alcoholic substrates such as, hydrocarbons [22]. The functional role of the AOxs from *A.terreus* is thus recognized as catalyst for the oxidation of the intermediate alcohol substrates formed during the CYP450 catalyzed degradation of various hydrocarbon substrates [45].

Reports on the crystal structures of AOx enzymes from fungal sources are limited with PDB id 1VAO and 3FIM from *P. simplicissimum* [29] and *P. eryngii* [44], respectively are the most prominent submissions. Both these crystal structures revealed significant dissimilarities between their 3D structures as well as in their amino acid sequence content. Multiple sequence alignment of *A.terreus* rAOx with the above aryl AOxs from these lignin degrading strains revealed significant sequence diversity. The cDNA sequence of the aryl AOx from *A.terreus* deduced by us consisted of 666 amino acids, whereas, the widely studied aryl AOxs from *P. pulmonarius*, *P. eryngii* and *P*. *simplicissimum* consisted of 594, 593 and 560 amino acid residues, respectively [43], [44], [2]. The amino acid sequence identity (using NCBI BLAST) of *A.terreus* AOx with other aryl AOxs from *P. pulmonarius*, *P. eryngii* and *P*. *simplicissimum* showed 27%, 25% and 37%, respectively. Even with the prevailing sequence variation, the predicted model of *A.terreus* rAOx showed significant structural homology with chain B of aryl AOx from *P. eryngii* (PDB id: 3FIM) [9] and its function was that of an aromatic AOx. The Ramachandran plot predicted our modeled protein to be stereo-chemically significant, thus increasing the authenticity of the *ab-initio* based 3D model. Further validations of our modeled 3D structure were proven through docking simulation studies with its co-factor FAD, which precisely predicted the conserved N-terminal binding region (Rossmann fold; GXGXXG motif, X = any amino acid residue) in our model. The docking also confirmed the β-α-β fold essential for non-covalent interaction with FAD. Function as predicted by I-TASSER was further validated through docking simulations carried out with our modeled rAOx along with four aromatic alcohols used in our kinetic studies. Based on MolDock scoring function the substrates were evaluated on the basis of its binding energies and was found to be consistent with the kinetics studies. The docking studies clearly demonstrated the close proximity of the active substrate binding site and the co-factor FAD binding site separated by a narrow funnel shaped cavity connecting the both (**data not shown**). It also confirmed that the active site lies in close vicinity of the FAD isoalloxazine ring. This kind of topology is conserved across all members of Glucose-Methanol-Choline (GMC) oxidoreductase family of proteins and is well reported [10], [16]. Presence of few conserved aromatic amino acid residues (Phe 98 and Tyr 55) near the FAD isoalloxazine ring and substrate binding site could be involved in π-π stacking interaction with FAD isoalloxazine ring, thus stabilizing the co-factor. Molecular modeling and docking results gave a visual insight into better understanding on the function and catalytic mechanism of this novel AOx enzyme.

The future objective of the current work is to elucidate the active side residues through x-ray crystallographic study. Notably, recombinant AOx isolated from fungal expression system are difficult to crystallize due to the microheterogeniety of carbohydrate moieties and other contaminants which are also co-purified with the target protein [35]. Moreover, fungal expression leads to glycosylation of the protein, which significantly increases the x-ray diffraction [44] leading to poor resolution of the crystal structure. Considering the above facts, *E.coli* has been considered for cloning and expression of the AOx gene from *A.terreus*. Moreover, in bacterial system the cloning strategy is straightforward and less time consuming as the protocol on heterologous expression in bacterial system and associated purification steps of the expressed protein has been substantially improved since last decade.

The reconstitution of the co-factor FAD with the apo-protein is a critical step in achieving functionally active protein. The refolding of the apo-AOx was achieved under alkaline and moderately low temperature conditions [35]. However, to achieve high recovery of the reconstituted enzyme and to avoid the technical difficulty paused by the refolding buffer in purifying the histidine tagged refolded protein through affinity column, the method was partially modified. Accordingly, the refolding step was performed after purifying the apo-AOx from the inclusion bodies through Ni^2+^- affinity column. A significant recovery of the functionally active rAOx from inclusion bodies was achieved and the fluorescence data confirmed the successful incorporation of cofactor FAD with the protein matrix. CD study of the rAOx was carried out in presence of glycerol (a critical component of the refolding buffer), which prevented off-pathway aggregation and acted as an osmolyte thus conferred structural stability to our native refolded protein.

Comparing MALDI-TOF/TOF mass of apo-AOx with SDS-PAGE revealed a loss of ∼1.6 kDa (mass of N-terminal and C-terminal 6× Histidine tag combined) possibly due to the poor ionization of trypsinized N- and C- terminal peptide fragments and poor detection at detector surface. The *p*I (6.5±0.1) of the apo-AOx was found to be more basic compared to the *p*I of other reported aryl rAOx expressed in *E.coli* [35]. The reason is attributed to the presence of higher basic amino acid residues that constituted ∼17% of the total amino acids present in the AOx protein.

## Conclusion

The sequencing, cloning, heterologous expression, purification, *in vitro* activation, biophysical characterization and *in silico* structure – function studies of a novel aryl AOx gene from a wild type isolate of hydrocarbon degrading *A.terreus* MTCC6324 are accomplished. This is the first report on the characterization of a full length coding nucleotide sequence of AOx from filamentous fungi following a combined proteomics and genomics approach. The significant sequence variation of the characterized *A.terreus* rAOx protein from the prevailing AOxs demonstrated the novelty of the protein even though functionally it is closer to the reported aryl AOx from few lignin degrading fungi. The work will advance the knowledge on the characteristics of AOx from filamentous fungi and will open the scope for production of the functionally active enzyme through recombinant DNA technology.

## Supporting Information

Figure S1 **Checking the orientation of the cloned PCR fragments (PCR 1 & 2) in TA cloning vector through double restriction digestion with** ***Nde*** **I and** ***Bgl*** **II, respectively.** Lane M represents a wide range DNA molecular weight marker, lane L1 shows the double digested fragment pattern of PCR 2 releasing ∼1278 bp (having the 3′ stop codon of AOx gene), lane L2 shows the double digested fragment pattern of PCR 1 releasing ∼3752 bp fragment (having the 5′ start codon of AOx gene).
(TIF)

Figure S2 **Confirmation of full length clone of AOx in pET28a (+) with double digestion.** Lane M is a wide range DNA molecular weight marker, lane L1 shows an undigested cloned pET28a(+) plasmid, lane L2 shows the clone confirmation of pET28a(+) subcloned AOx gene with flanking *EcoR*I and *Hind*III restriction site at its 5′ and 3′ end, respectively by double digestion with respective enzymes. Fragment release at ∼2001 bp and vector backbone at ∼5369 bp confirmed the clone. The agarose gel concentration was 0.8% and stained with ethidium bromide.
(TIF)

Figure S3 **Optimization of rAOx expression in** ***E.coli*** **BL21 (DE3) for IPTG concentration and time of induction.** Lane M is a protein molecular weight marker, Lane L1–L2, L3–L4 and L5–L6 are the supernatant– pellet fractions loaded adjacent to each other for 0, 0. 25 and 0.5 mM IPTG induction, respectively. The expression was monitored for 0 h, 4 h and 8 h for its optimal time of induction corresponding to maximum over-expression. The over expressed rAOx protein of ∼76 kDa(marked with a red dot) was observed in the pellet fraction of 4 h and 8 h IPTG induced cell lysate.
(TIF)

Figure S4 **Optimization of temperature parameter for rAOx over-expression in** ***E.coli*** **.** Lane M is a molecular weight marker, Lane L1–l8 are the supernatant and pellet fractions loaded alternatively for 15°C, 20°C, 25°C and 30°C induction temperature respectively at a constant shaking condition for 4 h and 0.25 mM.IPTG concentration. Over-expressed protein band as inclusion bodies are marked against red dots.
(TIF)

Figure S5 **MALDI –TOF/TOF analysis of rAOx.** Protein pilot database search of spot based MS data of trypsin digested purified rAOx is shown above. Matched peptides after MS/MS are highlighted in red uppercase single letter amino acid code having 28% sequence coverage with hypothetical un-reviewed amino acid sequence of AOx from *A.terreus* NIH2624. The mass of apo-rAOx was observed to be 74,614 Da.
(TIF)

Figure S6 **Determing the isoelectric point (** ***p*** **I) of rAOx through 2D electrophoresis and Zeta potential studies.** (**A**). Isoelectric point of rAOx as determined using 2D electrophoresis on immobiline dry strip pH 3.0 to 10.0 linear gradient. p*I* of rAOx is shown in black arrow head. The protein spot is also shown as a sharp peak in 3D box of the zone marked on the gel (side panel). (**B**). Zeta potential curve of rAOx with varying pH in range from pH 5.7 to 7.2. The point where the curve crosses the zero potential is shown as black dashed drop line on x-axis and was calculated to be 6.52.
(TIF)

Figure S7 **The active substrate binding site of the predicted holoenzyme rAOx model.** Active site residues surrounding *ρ*-methoxybenzyl alcohol (shown as thick stick CPK model) as the docked ligand near FAD isoalloxazine ring (shown as thick stick CPK model) at an atomic search radius of 6.0 Å is shown in the figure. Residues Tyr55, Pro56, Phe98, Asn354 were found to be conserved in all the docking results performed in our studies and are pointed out in the picture with a red arrow-head.The image is generated using Molegro Virtual Docker version 4.0.2 (CLC bio-Qiagen company).
(TIF)

Table S1 Oligonucleotide PCR primers used in this study.
(DOC)
