# Supplementary figures and images for: Molecular Characterization and Expression of a Novel Alcohol Oxidase from Aspergillus terreus MTCC6324
Source: PLoS One. 2014 Apr 21;9(4):e95368. doi: 10.1371/journal.pone.0095368 (PMC3994049; doi:10.1371/journal.pone.0095368)

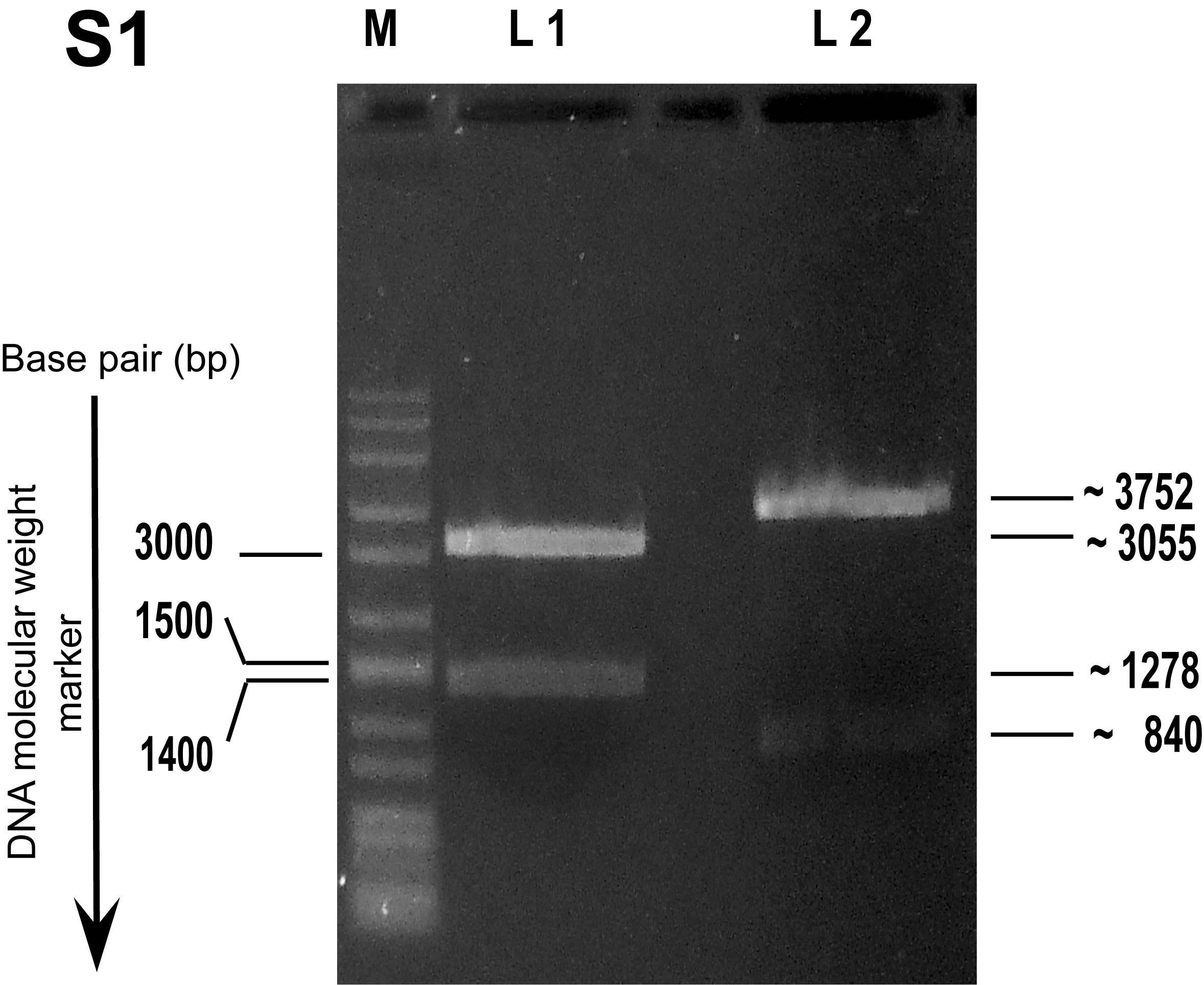

Supplement: Figure S1 — Checking the orientation of the cloned PCR fragments (PCR 1 & 2) in TA cloning vector through double restriction digestion with Nde I and Bgl II, respectively. Lane M represents a wide range DNA molecular weight marker, lane L1 shows the double digested fragment pattern of PCR 2 releasing ∼1278 bp (having the 3′ stop codon of AOx gene), lane L2 shows the double digested fragment pattern of PCR 1 releasing ∼3752 bp fragment (having the 5′ start codon of AOx gene). (TIF) [file pone.0095368.s001.tif]

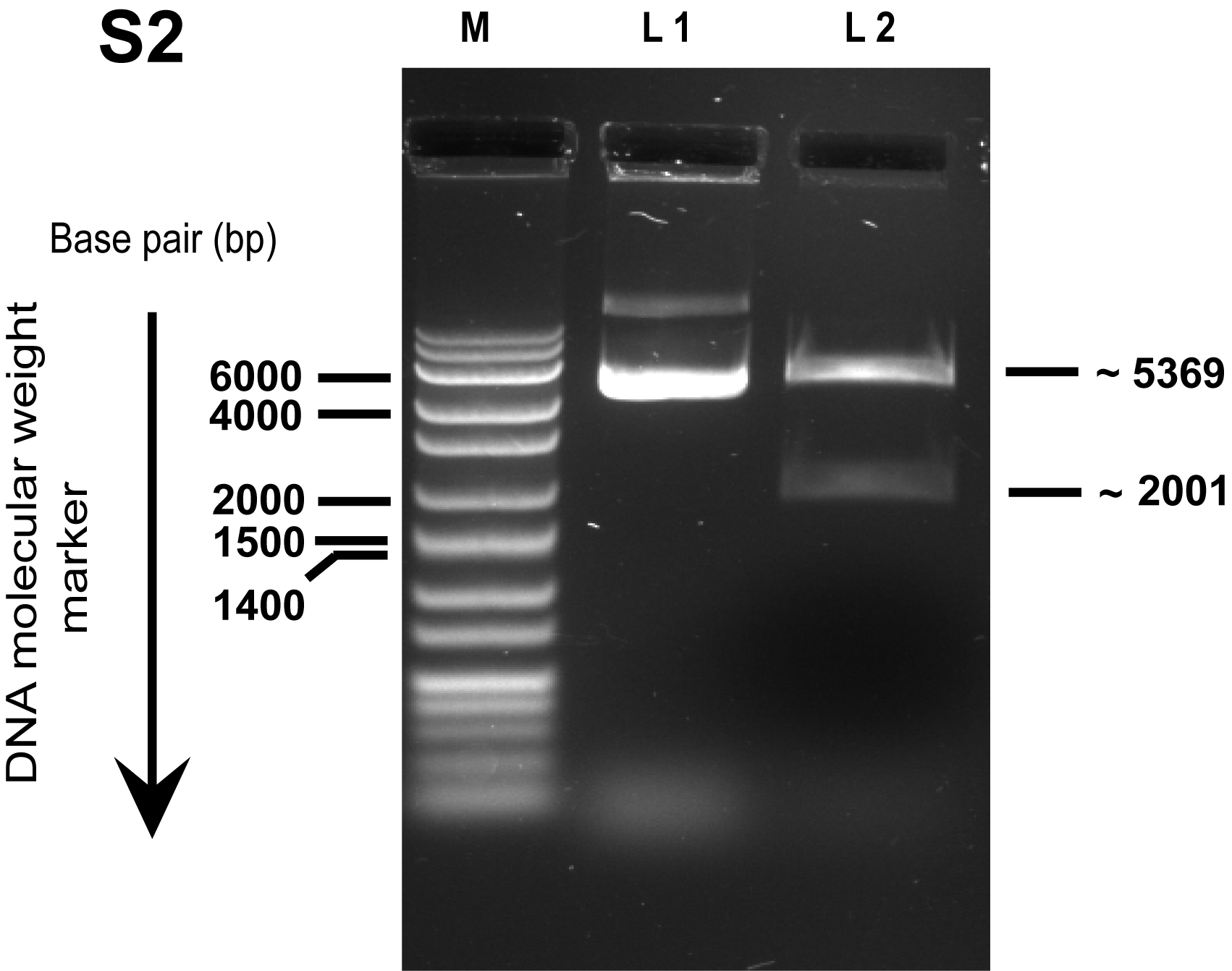

Supplement: Figure S2 — Confirmation of full length clone of AOx in pET28a (+) with double digestion. Lane M is a wide range DNA molecular weight marker, lane L1 shows an undigested cloned pET28a(+) plasmid, lane L2 shows the clone confirmation of pET28a(+) subcloned AOx gene with flanking EcoRI and HindIII restriction site at its 5′ and 3′ end, respectively by double digestion with respective enzymes. Fragment release at ∼2001 bp and vector backbone at ∼5369 bp confirmed the clone. The agarose gel concentration was 0.8% and stained with ethidium bromide. (TIF) [file pone.0095368.s002.tif]

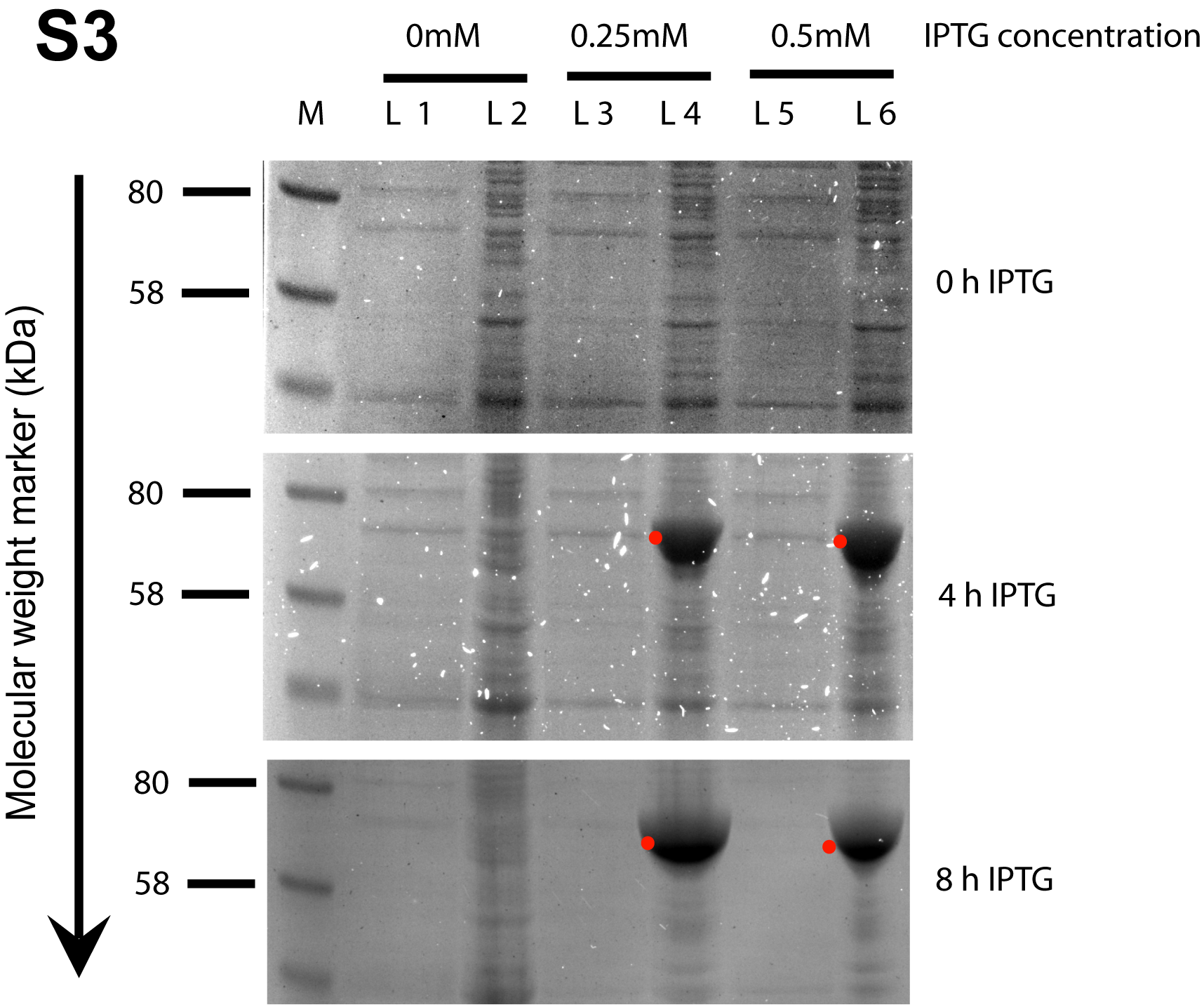

Supplement: Figure S3 — Optimization of rAOx expression in E.coli BL21 (DE3) for IPTG concentration and time of induction. Lane M is a protein molecular weight marker, Lane L1–L2, L3–L4 and L5–L6 are the supernatant– pellet fractions loaded adjacent to each other for 0, 0. 25 and 0.5 mM IPTG induction, respectively. The expression was monitored for 0 h, 4 h and 8 h for its optimal time of induction corresponding to maximum over-expression. The over expressed rAOx protein of ∼76 kDa(marked with a red dot) was observed in the pellet fraction of 4 h and 8 h IPTG induced cell lysate. (TIF) [file pone.0095368.s003.tif]

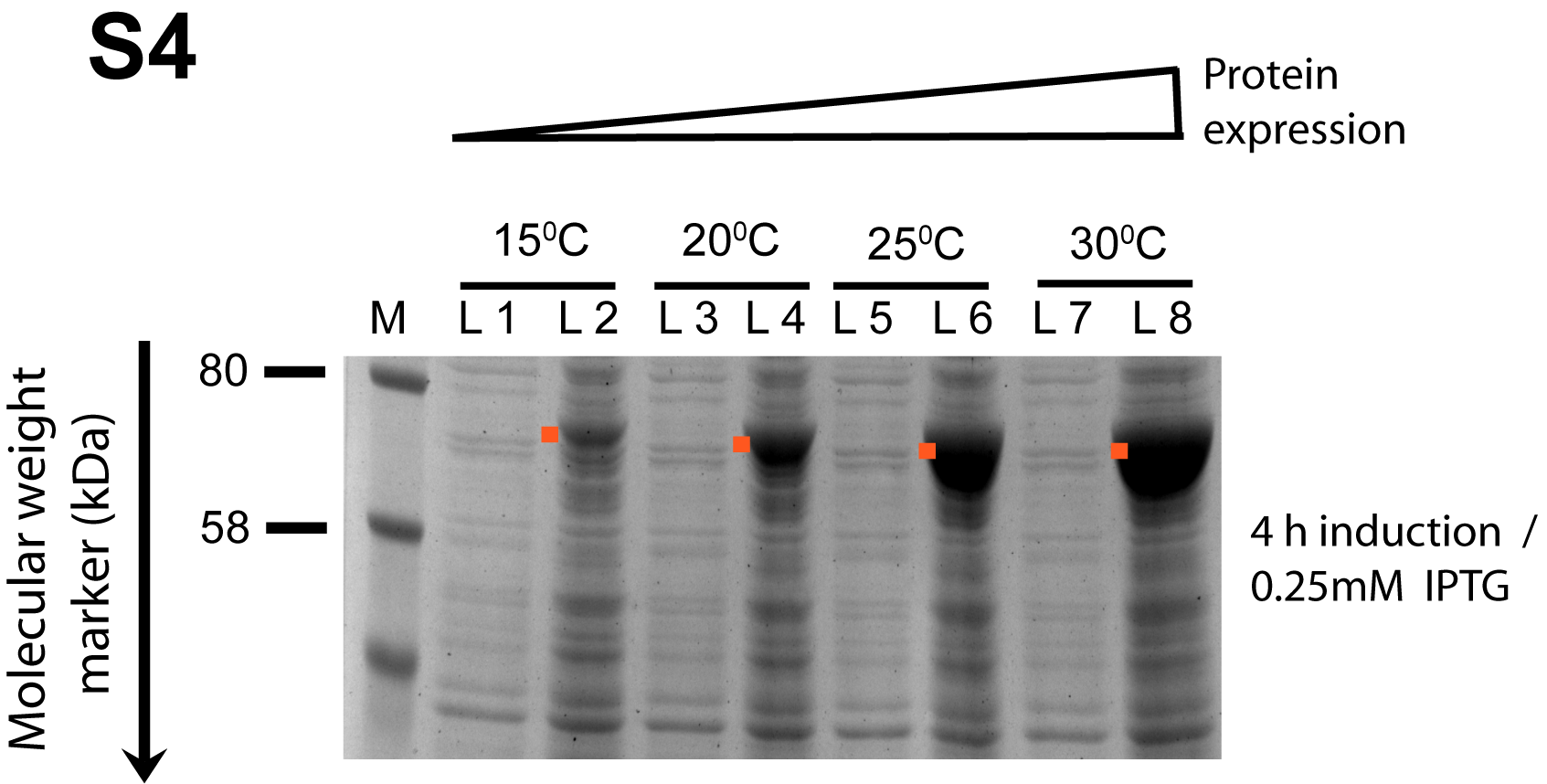

Supplement: Figure S4 — Optimization of temperature parameter for rAOx over-expression in E.coli . Lane M is a molecular weight marker, Lane L1–l8 are the supernatant and pellet fractions loaded alternatively for 15°C, 20°C, 25°C and 30°C induction temperature respectively at a constant shaking condition for 4 h and 0.25 mM.IPTG concentration. Over-expressed protein band as inclusion bodies are marked against red dots. (TIF) [file pone.0095368.s004.tif]

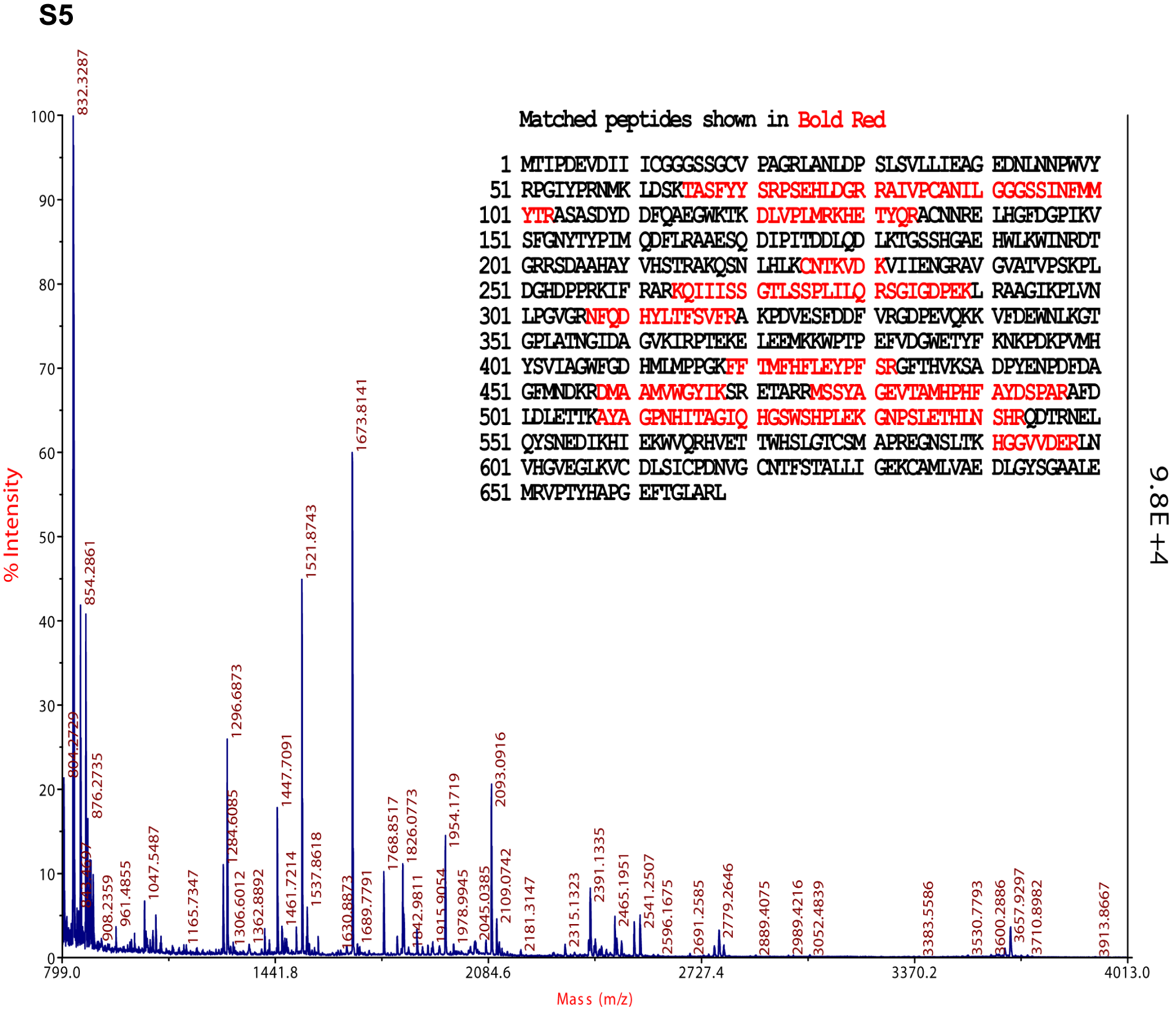

Supplement: Figure S5 — MALDI –TOF/TOF analysis of rAOx. Protein pilot database search of spot based MS data of trypsin digested purified rAOx is shown above. Matched peptides after MS/MS are highlighted in red uppercase single letter amino acid code having 28% sequence coverage with hypothetical un-reviewed amino acid sequence of AOx from A.terreus NIH2624. The mass of apo-rAOx was observed to be 74,614 Da. (TIF) [file pone.0095368.s005.tif]

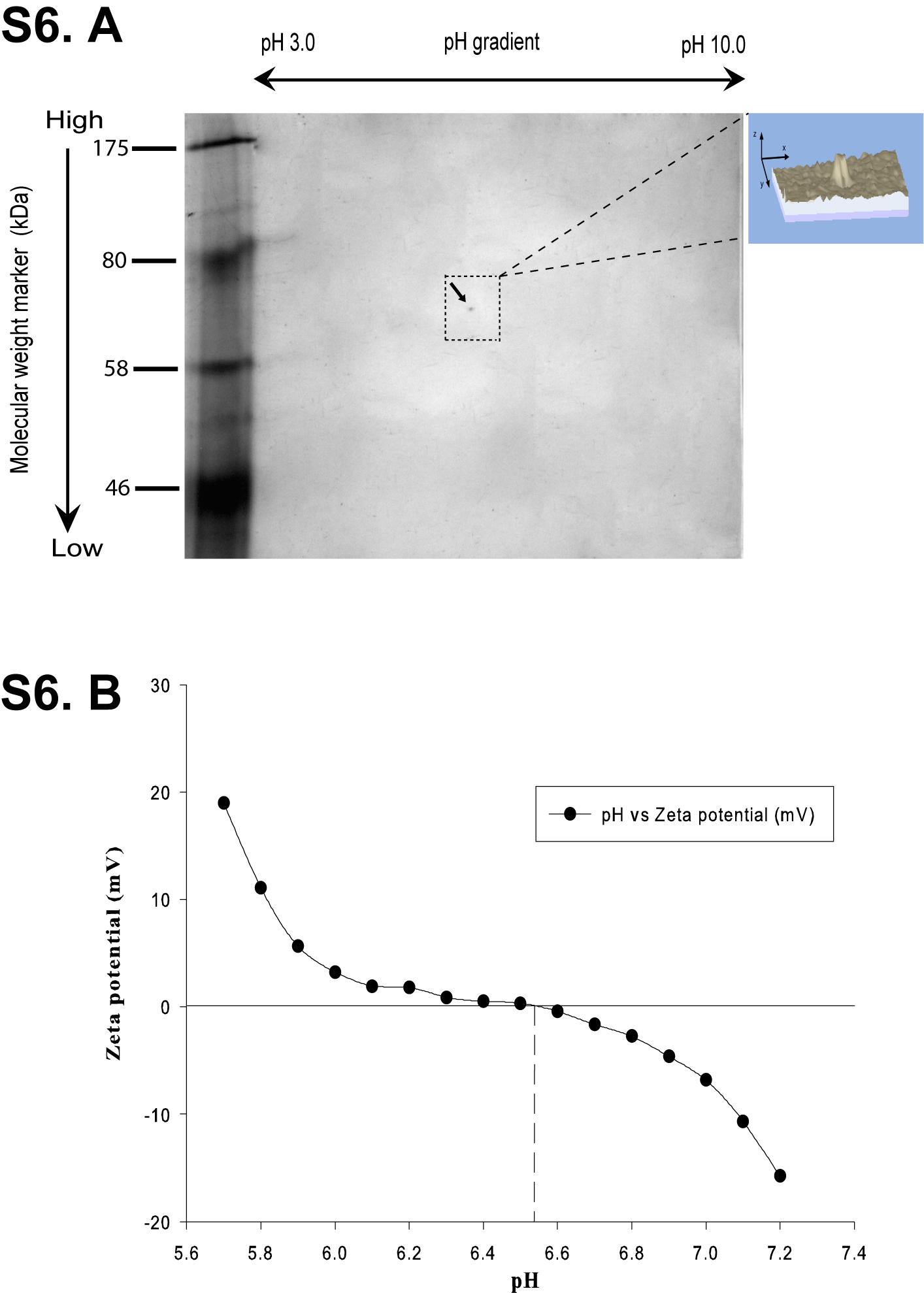

Supplement: Figure S6 — Determing the isoelectric point ( p I) of rAOx through 2D electrophoresis and Zeta potential studies. (A). Isoelectric point of rAOx as determined using 2D electrophoresis on immobiline dry strip pH 3.0 to 10.0 linear gradient. pI of rAOx is shown in black arrow head. The protein spot is also shown as a sharp peak in 3D box of the zone marked on the gel (side panel). (B). Zeta potential curve of rAOx with varying pH in range from pH 5.7 to 7.2. The point where the curve crosses the zero potential is shown as black dashed drop line on x-axis and was calculated to be 6.52. (TIF) [file pone.0095368.s006.tif]

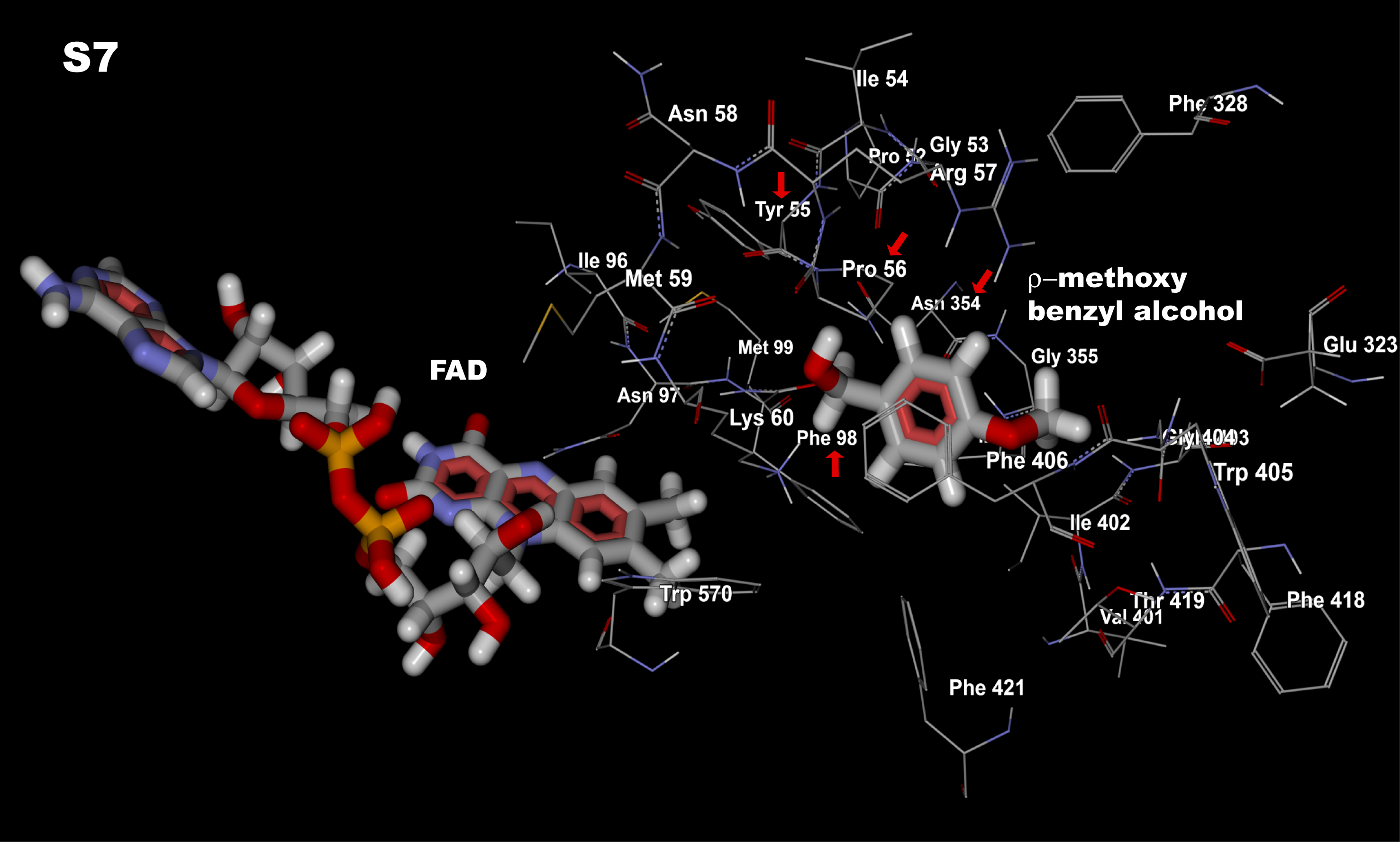

Supplement: Figure S7 — The active substrate binding site of the predicted holoenzyme rAOx model. Active site residues surrounding ρ-methoxybenzyl alcohol (shown as thick stick CPK model) as the docked ligand near FAD isoalloxazine ring (shown as thick stick CPK model) at an atomic search radius of 6.0 Å is shown in the figure. Residues Tyr55, Pro56, Phe98, Asn354 were found to be conserved in all the docking results performed in our studies and are pointed out in the picture with a red arrow-head.The image is generated using Molegro Virtual Docker version 4.0.2 (CLC bio-Qiagen company). (TIF) [file pone.0095368.s007.tif]
