# Supplementary material for: Molecular Characterization and Expression of a Novel Alcohol Oxidase from Aspergillus terreus MTCC6324
Source: PLoS One. 2014 Apr 21;9(4):e95368. doi: 10.1371/journal.pone.0095368 (PMC3994049; doi:10.1371/journal.pone.0095368)
Supplement: Table S1 — Oligonucleotide PCR primers used in this study. (DOC) [file pone.0095368.s008.doc]

**Table S1.**

| **Sl No** | **Name** | **Sequence (5’- 3’)** |
| --- | --- | --- |
| 1 | GAPDH-F | CAAGGTCATCCATGACAACTTTG |
| 2 | GAPDH-R | GTCCACCACCCTGTTGCTGTAG |
| 3 | AOX-FP1 | ATGACTATTCCAGACGAAGTCGACA |
| 4 | AOX-RP1 | TGAGACCAGGATCCATGCTGGAT |
| 5 | AOX-FP2 | ACAGTCCCCTCCAAGCCGCT |
| 6 | AOX-RP2 | TTACAGTCGAGCAAGCCCAGTAAACTC |
| 7 | AOX-pET28a-F | GCC**GAATTC**ATGACTATTCCAGACGAAG |
| 8 | AOX-pET28a-R | CGC**AAGCTT**CAGTCGAGCAAGCCCAGT |
